# Reporting practices and impact of withdrawal of life-sustaining treatment on outcomes in acute brain injury clinical trials: a literature review and simulation study

**DOI:** 10.1186/s13054-026-05929-7

**Published:** 2026-03-08

**Authors:** Shaurya Taran, Jeffrey M. Singh, Christopher J. Yarnell, Victoria A. McCredie, Damon C. Scales, Niall D. Ferguson, Kuan Liu, Neill K. J. Adhikari

**Affiliations:** 1 Department of Medicine, University Health Network, Toronto, ON Canada; 2 Interdepartmental Division of Critical Care Medicine, University of Toronto, Toronto, ON Canada; 3 Institute of Health Policy, Management, and Evaluation, University of Toronto, Toronto, ON Canada; 4 Department of Critical Care Medicine and Research Institute, Scarborough Health Network, Toronto, Canada; 5 Department of Critical Care Medicine, Sunnybrook Health Sciences Centre, Toronto, Canada; 6 Toronto General Hospital Research Institute, Toronto, ON Canada; 7 Toronto Western Hospital Office 411-L, 2nd Floor McLaughlin 399 Bathurst St, Toronto, M5T 2S8 ON Canada

**Keywords:** Acute brain injury, Withdrawal of life-sustaining treatment, Randomized clinical trials, Bias

## Abstract

**Background:**

Withdrawal of life-sustaining treatment (WLST) is common in clinical trials of patients with acute brain injuries (ABI), but current reporting practices and impact on trial-reported findings are unclear. We evaluated reporting practices of WLST in contemporary clinical trials of patients with ABI and quantified the magnitude of bias on treatment effect estimates in hypothetical trials.

**Methods:**

We conducted a literature review of contemporary ABI randomized clinical trials and a simulation-based analysis. In the literature review, we included two-arm, randomized, superiority trials of adults with ABI (traumatic brain injury, intracranial hemorrhage, subarachnoid hemorrhage, ischemic stroke, or post–cardiac arrest brain injury) published in 10 high-impact journals from January 1, 2015 to December 19, 2024. We extracted WLST characteristics including frequency, timing, reasons, and neuro-prognostication criteria. In the simulation-based analysis, we evaluated the impact of WLST misclassification—defined as WLST occurring in patients who could have survived with a good neurological outcome—on observed treatment effects. For each scenario, we estimated the observed treatment effect after misclassification and calculated bias as the difference between observed and true treatment effects. We assessed both blinded and unblinded trials and binary and ordinal neurologic outcomes.

**Results:**

Among 69 trials included in the literature review, 17 trials (24.6%) reported WLST frequency, 9 (13.0%) timing, 10 (14.5%) reasons, and 7 (10.1%) standardized neuro-prognostication criteria. In simulations of blinded trials, WLST misclassification consistently attenuated observed treatment effects. Increasing the fraction of misclassified WLST events led to progressively greater bias, making beneficial treatments appear less effective and harmful treatments appear less harmful. In unblinded trial simulations, the direction of bias varied by the magnitude of the true treatment effect and degree of misclassification. Findings were similar for binary and ordinal neurologic outcomes. Across all simulations, WLST misclassification reversed statistical conclusions in a median of 22.1% (interquartile range 17.4–32.4%) of trials.

**Conclusions:**

WLST is poorly reported in contemporary ABI trials. Misclassification of WLST-related deaths leads to important bias in trial-reported treatment effects, potentially yielding underpowered studies and erroneous trial conclusions. Standardized, transparent WLST reporting is essential to strengthen ABI trial design and interpretation.

**Supplementary Information:**

The online version contains supplementary material available at 10.1186/s13054-026-05929-7.

## Introduction

Most deaths in critically ill patients with acute brain injuries (ABI) occur after a decision is made to withdraw life-sustaining treatment (WLST) [1–3]. While many decisions are associated with devastating injuries and little chance of recovery, some patients in whom treatment is withdrawn could have achieved acceptable neurologic outcomes if treatment had continued [4, 5]. WLST is also associated with factors beyond neurologic prognosis, such as patient demographics (e.g., chronic conditions, religious beliefs, health insurance status); clinician factors; and center, country, or region of management [6–11].

These findings have critical implications for the interpretation of treatment effects in ABI clinical trials. Given the high incidence of WLST, recovery potential of some patients, and impact of non-clinical factors on withdrawal decisions, it is possible that outcomes in some trials could reflect WLST patterns rather than intrinsic treatment effects. Comprehensive reporting of WLST characteristics can help clarify the extent to which treatment effects may be biased [12, 13]; however, this information is rarely reported in trials [14]. A systematic review of 41 traumatic brain injury trials published between 2002 and 2015 found that only 20% reported WLST frequency, 15% reported WLST timing, and 12% reported reasons for withdrawal [15]. Whether WLST reporting practices are different in contemporary trials of patients with other ABI conditions remains unknown. Moreover, quantifying the impact of WLST is crucial to appreciating the direction and magnitude of potential bias in clinical trials.

Therefore, we (1) performed a literature review to characterize WLST reporting practices in contemporary ABI trials, and (2) conducted a simulation study to evaluate the magnitude of WLST-related bias on outcomes in a series of hypothetical trials. We hypothesized that WLST is infrequently reported in contemporary ABI trials and that its occurrence would distort the direction and magnitude of reported treatment effects.

## Methods

### Literature review

We searched PubMed from January 1, 2015 through December 19, 2024 for randomized clinical trials published in the following ten journals: *Lancet*, *Lancet Neurology*, *Lancet Respiratory Medicine*,* JAMA*,* JAMA Neurology*, *Neurology*, *American Journal of Respiratory and Critical Care Medicine*, *Intensive Care Medicine*,* British Medical Journal*, and *New England Journal of Medicine.* Journal selection aimed to (1) capture high-impact journals most likely to publish practice-changing ABI trials, and (2) reflect prevailing WLST reporting standards in the field. Details of the search and article screening are provided in eSupplement1. We included trials conducted in patients with ABI, defined as traumatic brain injury, subarachnoid hemorrhage, intracranial hemorrhage, acute ischemic stroke, or post-cardiac arrest brain injury. We extracted information on clinical condition, sample size, intervention characteristics, primary outcome, key findings, and the following characteristics related to WLST: frequency of events, timing of decisions, reasons for withdrawal, and whether neuro-prognostication before WLST was standardized. Trial-level information is presented in tables and summarized using descriptive statistics.

### Simulations

**Fig. 1:**
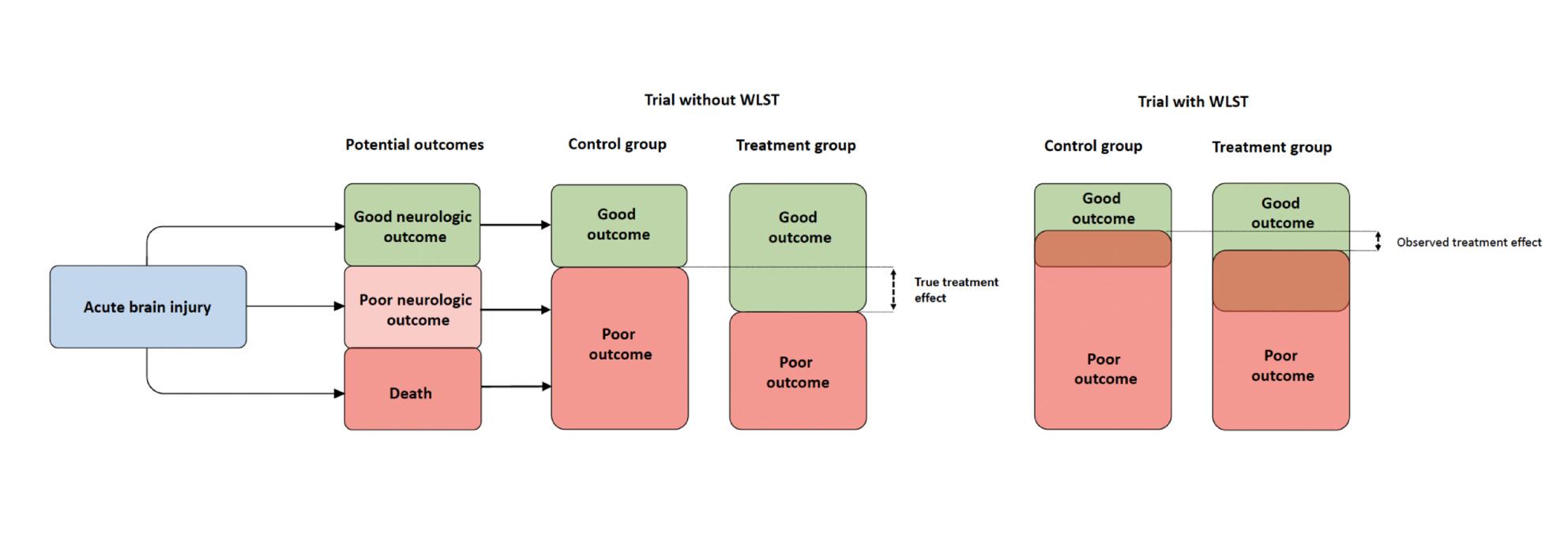
This figure outlines the potential mechanism of bias related to WLST in a two-arm, superiority randomized clinical trial of ABI patients where the outcome is expressed as a binary variable (poor outcome = death or severe neurologic disability; good outcome = moderate neurologic deficits or better). In a trial with no WLST, any difference in outcomes between the treatment and control groups is completely due to the treatment (i.e., the true treatment effect), which in the figure is represented by the difference in poor outcome probabilities between groups. However, in real-world trials, WLST misclassifications could cause some withdrawal events to occur in patients who could have otherwise survived with an acceptable (or better) neurologic outcome. Since WLST decisions nearly always result in death, some individuals will be misclassified from a good outcome to a poor outcome (denoted as the brown shaded areas in the trial with WLST). This changes the balance of outcomes between groups, potentially causing the observed treatment effect to be different than the true treatment effect. Depending on the proportion of misclassifications that occur in the treatment and control groups, the observed treatment effect could be larger or smaller than the true treatment effect. ABI trials could be strengthened by rigorous WLST practices to ensure that misclassifications remain small, otherwise findings may be susceptible to bias. WLST, withdrawal of life-sustaining treatment

Using study-level inputs from our literature review, we simulated a series of hypothetical randomized trials to estimate the impact of WLST on trial-reported treatment effects. Simulations were used to explore general patterns of bias across a range of realistic trial designs. We used study-level (rather than patient-level) data to make the simulations computationally feasible and to focus on trial-level dynamics that apply broadly across ABI research; we did not intend to reproduce any single study. The putative mechanism of bias due to WLST is illustrated in Fig. 1, the simulation process is shown in Table 1, and technical details are provided in eSupplement2 in the appendix. Simulations were performed using R 4.0.2 (R Foundation for Statistical Computing, Vienna, Austria).

In the main analysis, simulated trials measured a binary neurologic outcome (poor=death or severe neurologic disability; good=moderate neurologic disability or better). In additional analyses, simulated trials measured ordinal neurologic outcomes. In all analyses, we assumed that (1) all WLST events resulted in death [16], thereby masking the outcome that would have occurred in the absence of a withdrawal decision, and (2) that in a proportion of cases, WLST was performed in patients who could have survived with a good neurologic outcome. We refer to the fraction of WLST done in such patients (who died due to WLST rather than surviving to see a good outcome) as the *misclassified fraction*.

### Estimating bias introduced by WLST misclassification 

We used simulations to compare the *true treatment effect* and the *observed treatment effect*. The true treatment effect was defined as the effect of treatment on neurologic outcome from a trial with no WLST misclassification (i.e., any difference in outcome is solely due to treatment). The observed treatment effect was defined as the effect of treatment on neurologic outcome after WLST-related misclassification. The difference between the true and observed treatment effects was defined as *bias*. 95% Confidence intervals (CIs) for the observed treatment effect and bias were derived non-parametrically across 1,000 iterations of each simulation. We also captured sampling variability inherent to the simulation process using Monte Carlo standard errors [17]. The overall goal of this analysis was to answer the following question related to the general contextual effect of WLST: If the same trial were to be repeated many times, what would be the average effect of WLST misclassification on treatment estimates across all trials?

To examine the contextual effect of WLST across different hypothetical trials, we reported the observed treatment effect and bias for a range of misclassified fractions and true treatment effects. For the latter, we studied effective treatments (i.e., treatment leads to fewer poor neurologic outcomes in the treated group), neutral treatments (i.e., treatment leads to no difference in neurologic outcomes between groups), and harmful treatments (i.e., treatment *increases* poor neurologic outcomes in the treated group), as described in Table 1.

We also estimated the proportion of simulated trials in which WLST misclassification changed the statistical interpretation of results from significant to non-significant or vice versa, based on whether the 95%CI of individual trials included the null value. The goal of this analysis was to answer the following question related to the individual, trial-level effect of WLST: If the same trial were to be repeated many times, in what proportion of trials would conclusions about the treatment change?

### Estimating impact of WLST misclassification according to study design

To determine the extent that blinding or unblinding could impact trial results, we studied two scenarios: one in which the misclassified fraction was the same between the treatment and control groups (clinicians are blinded to the intervention), and another in which the misclassified fraction was 20% lower in the treatment group compared to the control group (clinicians are unblinded and expect a benefit from the intervention). This proportion was specified a priori as a scenario analysis to explore directional sensitivity to plausible WLST imbalances rather than to represent empirically derived estimates of clinician behavior.

### Additional analyses

Several additional analyses were done. First, we repeated simulations for trials that measured neurologic outcome on an ordinal scale. We conducted two sets of simulations using ordinal neurologic outcome probabilities from a blood transfusion trial that showed modest clinical benefit [18], and a thrombectomy trial that showed significant clinical benefit [19]. We then applied different misclassified fractions and compared the trial-reported proportional odds ratios to the odds ratios obtained after WLST misclassification. Second, we performed simulations to evaluate the impact of a neutral and harmful treatment on an ordinal neurologic outcome. Third, in an exploratory analysis under simplified assumptions, we examined a statistical approach to attenuate bias. We applied inverse probability of censoring weighting (IPCW) [20] to estimate the true treatment effect in a subset of simulations, expecting this method to partially mitigate WLST-related bias. Fourth, we performed a post-hoc analysis for a subset of effective treatments in which we evaluated a 40% lower misclassified fraction in the treated (vs. untreated) group, reflecting strong belief in treatment efficacy within unblinded trials. Additional details are given in eSupplement2.

## Results

### Literature review

The search identified 607 trials, of which 69 were included. Table 2 summarizes key aspects of the trials; additional characteristics are presented in eTables1-2. Most trials were in patients with ischemic stroke (*n* = 27; 39.1%) and cardiac arrest (*n* = 19; 27.5%). Median (IQR) sample size was 408 (253-1,063; range, 50 − 23,711). Most trials were neutral for their primary endpoint (*n* = 37; 53.6%). Of the 30 trials with a positive primary endpoint, the majority were in patients with ischemic stroke (*n* = 17).

Overall, 17 trials (24.6%) reported frequency of WLST (Table 2). After excluding ischemic stroke trials, WLST was reported in 15/42 (35.7%) of trials. WLST frequency was reported in 11/19 (57.9%) cardiac arrest trials, 2/27 (7.4%) ischemic stroke trials, 2/7 (28.6%) intracranial hemorrhage trials, and in 2/9 (22.2%) traumatic brain injury trials. Neither of the two SAH trials and none of the four trials in heterogeneous ABI populations reported WLST frequency. Among trials where WLST frequency was reported, the median WLST fraction as a proportion of patients enrolled was 21.7 (IQR 9.5–32.2)%. Timing of WLST was reported in 9/17 trials (52.9%), reasons for WLST were reported in 10/17 trials (58.8%), and neuro-prognostication before WLST was standardized in 7/17 trials (41.2%); in all cases, these data were only reported in cardiac arrest trials (eTable3 in appendix). Overall, WLST was the most common cause of death in trials where this information was recorded, with a median of 65.7 (IQR 35.9–77.8)% of deaths being preceded by a WLST decision.

**Table 1:** Steps in the simulation design

Objective	Design process	Outcome
Determine a range of plausible treatment effect sizes	We used the absolute risk reduction (ARR) from the sample size calculation of each clinical trial as a measure of treatment effect. Five different treatment effect sizes were selected using quintiles of the ARR from all trials included in the literature review. To investigate additional real-world scenarios, in which many trials show no benefit and some show harm, we included ARR values of 0 (i.e., the treatment is of no benefit), and − 5 (i.e., the treatment is moderately harmful)	The following five treatment effect sizes were chosen, using quintiles of the ARR from published trials: 1.5%, 6%, 10%, 14%, 17%. Two additional ARRs of 0% and −5% were added, to give a total of seven treatment effects (five effective treatments, one neutral treatment, and one harmful treatment)
Determine a range of plausible baseline probabilities of the outcome of interest	We used the control group results from each clinical trial to select baseline probabilities of the outcome of interest in the simulation. These probabilities are assumed to represent outcomes among patients receiving the standard of care. Five different baseline outcome probabilities were selected using quintiles of the control group outcomes from trials included in the literature review. Because the treatment effect (above) is defined in terms of the ARR, we expressed control group outcomes in terms of a poor outcome	The following five baseline probabilities of a poor outcome were identified, using data from published trials with a binary primary outcome: 6%, 37%, 50%, 63%, 86%
Determine the sample size for each simulated trial	We created unique pairs of treatment effect and baseline outcome probability. Pairs that were clinically unlikely were removed (*see* eSupplement 2 in appendix for details). For remaining pairs, we derived a sample size for the trial assuming a two-sided alpha level of 0.05 and 80% power. To ensure clinical sensibility, we compared distributions of the resulting sample sizes with those from trials in the literature review	The sample size of trials in the simulations ranged from 188 to 17,544 patients, compared to 50 to 23,711 patients in trials in the literature review
Determine clinically appropriate misclassified fractions	We conducted literature reviews to determine what proportion of patients with a good neurologic outcome might undergo WLST (i.e., the “misclassified fraction”). Subsequent analyses were performed using a range of misclassified fractions to determine effects on trial conclusions	Misclassified fractions of 2%, 5%, 10%, and 20% were used across different simulations

## Blinded trial simulations

**Fig. 2:**
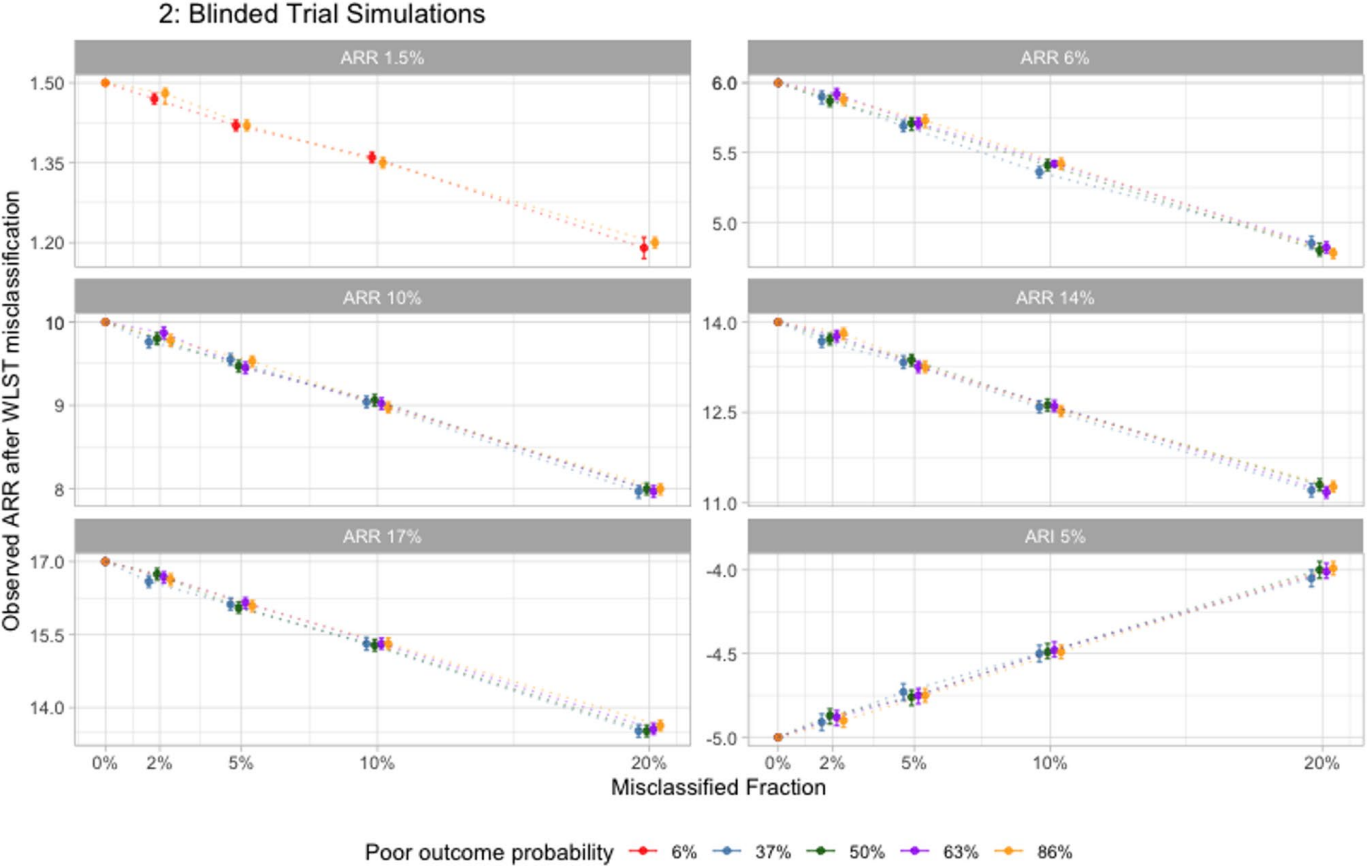
This figure shows the observed treatment effect from hypothetical blinded trials for different combinations of true treatment effect, baseline poor outcome probabilities, and WLST misclassified fractions. Each panel indicates, for a given true treatment effect (indicated in the grey shaded bars), the resulting observed treatment effect (represented by dots on the plot) at different levels of baseline illness severity (represented by different colors on the plot) and different misclassified fractions (indicated on the x-axis on each plot). The error bars around each dot represent Monte Carlo standard errors related to simulation-level variability. Blinding is simulated by holding the misclassified fraction constant between the treatment and control groups. In general, WLST misclassification causes the average observed treatment effect to attenuate relative to the true effect (i.e., the effect is drawn towards the null). This is true for both effective and harmful treatments and increases at higher misclassified fractions. ARI, absolute risk increase; ARR, absolute risk reduction; WLST, withdrawal of life-sustaining treatment

In blinded trials, the observed treatment effect attenuated across all levels of baseline illness severity and true treatment effect size, with higher misclassified fractions leading to larger bias (Fig. 2 and eTables4-9 in appendix). For effective treatments, the observed treatment effect was smaller than the true effect, whereas for a harmful treatment, the observed harm was smaller than the true harm. Reversal of statistical interpretation at the individual trial level was common: across 88 simulations of effective and harmful treatments (88,000 hypothetical trials), the median proportion of trials that showed reversal from effective (or harmful) to neutral was 23.5 (IQR 18.2–38.3)%. Reversal was also observed in the case of a harmful treatment, in which some simulations suggested the treatment was neutral (eTables4-9 in appendix).

### Unblinded trial simulations

**Fig. 3:**
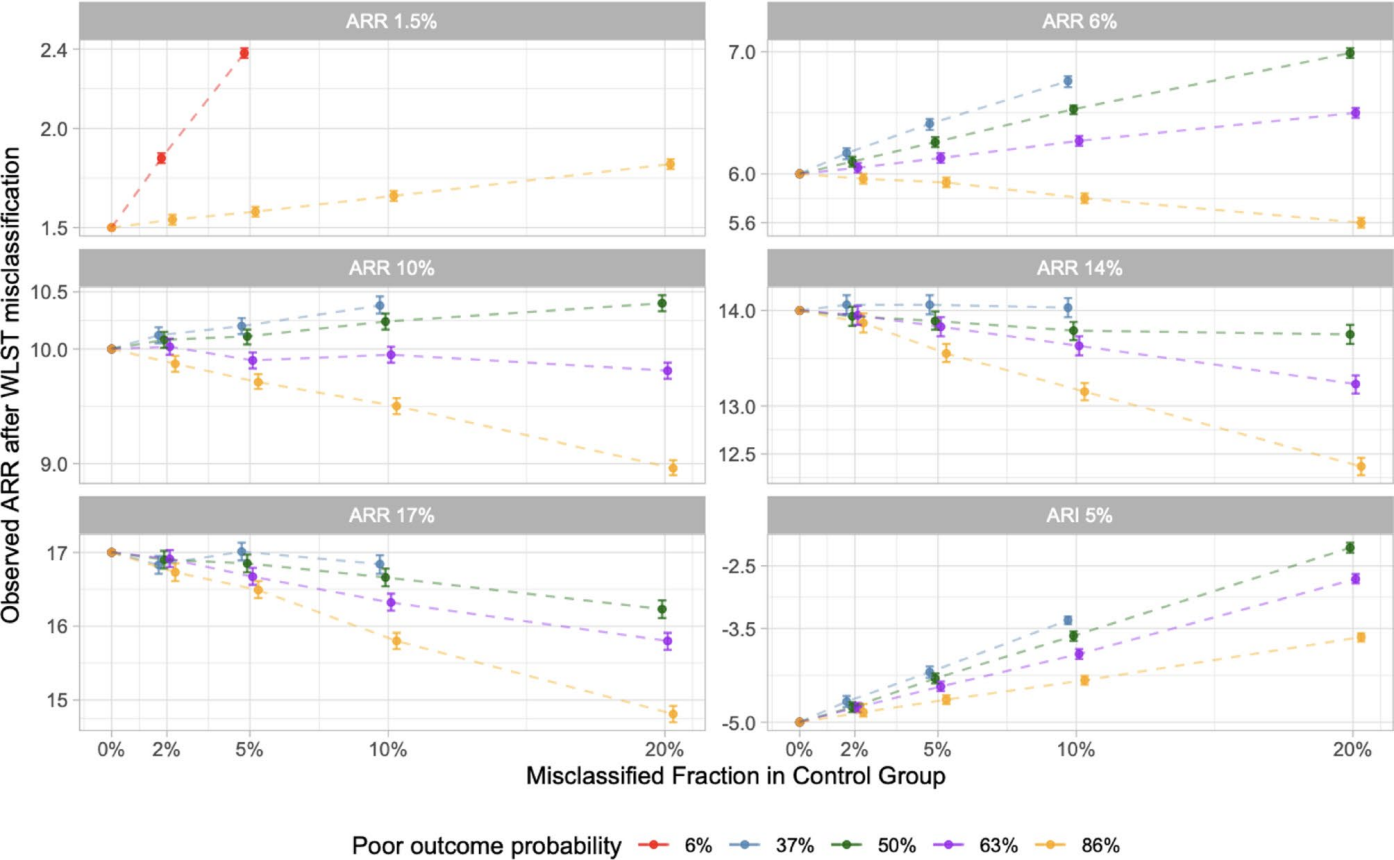
This figure shows the observed treatment effect from hypothetical unblinded trials for different combinations of true treatment effect, baseline poor outcome probabilities, and WLST misclassified fractions. Each panel indicates, for a given true treatment effect (indicated in the grey shaded bars), the resulting observed treatment effect (represented by dots on the plot) at different levels of baseline illness severity (represented by different colors on the plot) and different misclassified fractions in the control group (indicated on the x-axis on each plot). The error bars around each dot represent Monte Carlo standard errors related to simulation-level variability. Unblinding is simulated by lowering the misclassified fraction by 20% in the treatment group compared to the control group (e.g., in a trial with a misclassified fraction of 2% in the control group, the misclassified fraction in the treatment group would be 1.6%). As shown, the average observed treatment effect may be either larger or smaller than the true treatment effect depending on the particular characteristics of the trial). ARI, absolute risk increase; ARR, absolute risk reduction; WLST, withdrawal of life-sustaining treatment

In unblinded trials, the direction and magnitude of bias depended on the baseline illness severity of patients and true treatment effect size. In general, the benefit of modestly effective treatments appeared to increase as the misclassified fraction increased and the illness severity decreased (Fig. 3 and eTables10-14 in appendix). However, for treatments with a large effect size, the observed treatment effect remained close to, or smaller, than the true effect even at higher misclassified fractions. Across 66 simulations of effective treatments (66,000 hypothetical trials), reversal of results due to WLST misclassification occurred in a median of 19.3 (IQR 16.4–21.6)% of trials. For a neutral treatment, WLST misclassification shifted the point estimate of the observed treatment effect towards effectiveness. In the statistical reversal analysis, the neutral treatment appeared effective in a median of 4.7 (IQR 3.8–7.4)% of trials (*n* = 30 simulations; 30,000 trials); in a minority of cases, however, the reversal was in the direction of harm (eFigure1 and eTable15 in appendix). For a harmful treatment, the average observed harm appeared smaller than the true harm, and statistical reversal (i.e., harmful to neutral) was common (eTable16 in appendix).

**Table 2:** Characteristics of included trials

Variable	Overall, *n* = 69	Stroke, ^a^ *n* = 36	Cardiac arrest, *n* = 19	Traumatic brain injury, *n* = 9	Mixed ABI conditions, ^b^ *n* = 5
Outcome type, n (%)					
Binary	46 (66.7)	19 (52.8)	18 (94.7)	6 (66.7)	3 (60.0)
Ordinal	23 (33.3)	17 (47.2)	1 (5.3)	3 (33.3)	2 (40.0)
Sample size, median (IQR)	408 (253-1,063)	315 (205–737)	677 (398-2,452)	408 (339–742)	345 (325–850)
Intervention studied					
Medical	25 (36.2)	10 (27.8)	6 (31.6)	4 (44.4)	5 (100)
Surgical/interventional	26 (37.7)	22 (61.1)	2 (10.5)	2 (22.2)	0
Anesthesia	5 (7.2)	2 (5.6)	3 (15.8)	0	0
Hypothermia	6 (8.7)	0	5 (26.3)	1 (11.1)	0
System processes	4 (5.8)	2 (5.6)	2 (10.5)	0	0
Devices	3 (4.3)	0	1 (5.3)	2 (22.2)	0
Trial result ^c^					
Positive	30 (43.5)	21 (58.3)	5 (26.3)	2 (22.2)	2 (40.0)
Neutral	37 (53.6)	14 (38.9)	14 (73.7)	7 (77.8)	2 (40.0)
WLST characteristics					
Frequency reported	17 (24.6)	4 (11.1)	11 (57.9)	2 (22.2)	0
Timing reported	9 (13.0)	0	9 (47.4)	0	0
Reasons for WLST reported	10 (14.5)	0	10 (52.6)	0	0
Neuroprognostication before WLST standardized	7 (10.1)	0	7 (36.8)	0	0

This table indicates basic characteristics of the included trials. Additional details are reported in eTable 1 in the Supplement

^a^Included trials of patients with ischemic stroke (n=27), intracranial hemorrhage (n=7), and subarachnoid hemorrhage (n=2)

^b^Each trial included patients with more than one type of ABI condition

^c^Two trials in this review showed harm with the intervention and were not included in the column totals

ABI, acute brain injury; IQR, interquartile range

Across all blinded and unblinded simulations (199 simulations of 199,000 hypothetical trials), statistical interpretations changed in a median of 22.1 (IQR 17.4–32.4)% of trials. Worked examples of the impact of WLST-related misclassification across different trial scenarios are provided in Table 3.

**Table 3:** Worked examples of WLST-related misclassification in blinded and unblinded trials

True treatment effect (ARR)	Poor outcome probability	Misclassified fraction in control group	Misclassified fraction in treatment group	Observed treatment effect (ARR)	Interpretation
Blinded trial					
10%	50%	5%	5%	9.46%	WLST misclassification decreases perceived treatment efficacy, with greater bias at higher misclassified fractions. Effect does not depend on baseline poor outcome probability
10%	50%	10%	10%	8.97%	
10%	86%	10%	10%	8.93%	
Unblinded trial					
10%	50%	5%	4%	10.05%	WLST misclassification can either amplify or decrease the observed treatment effect. Direction and magnitude depend on baseline poor outcome probability
10%	50%	10%	8%	10.13%	
10%	86%	10%	8%	9.51%	

This table illustrates single-trial examples showing how WLST-related misclassification alters the apparent treatment effect. True treatment effects are expressed on the ARR scale and reflect the difference in poor neurologic outcome between control and treatment groups before WLST misclassification. Observed treatment effects (also on the ARR scale) reflect the apparent effect on trial outcomes after applying the specified misclassified fractions (proportion of patients who might have had a good outcome but instead died following WLST)

ARR, absolute risk reduction; WLST, withdrawal of life-sustaining treatment

### Additional analyses

Similar results were obtained in ordinal outcome analyses, with the transfusion and thrombectomy trials (two effective treatments) both showing attenuation of the observed treatment effect due to WLST misclassifications (eTable17 in appendix). The observed treatment effect of a neutral treatment was shifted towards benefit, while the effect of a harmful treatment was shifted towards the null (eTable18 in appendix). Bias from WLST-related misclassification was attenuated by IPCW (eTable19 in appendix). In the post-hoc analysis of effective treatments, bias was amplified when the misclassified fraction was 40% lower in the treated (vs. control group), implying strong belief in treatment (eTable20 in appendix). Impacts of WLST misclassification and potential mechanisms causing bias are summarized in Table 4.

**Table 4:** Potential impact of WLST in clinical trials

Scenario	Potential impact of WLST misclassification	Mechanism of bias
Blinded trial of an effective treatment	WLST misclassification could attenuate the observed treatment effect	Treatment increases the proportion of patients with a good outcome. Although the misclassified fraction is similar between groups, more patients in the treated group with a potentially good outcome are “switched” to a poor outcome. Therefore, the poor outcome probability appears more similar between groups, and the observed treatment effect is smaller
Blinded trial of an ineffective treatment	Likely minimal impact of WLST misclassification	Because the misclassified fraction is similar, the same proportion of patients are expected to be misclassified, and outcomes appear similar between groups. Bias is therefore unlikely
Blinded trial of a harmful treatment	WLST misclassification could attenuate the perception of treatment harm	Treatment reduces the chance of a good outcome. However, because misclassification is symmetrical, both groups look more similar, reducing the observed harm
Unblinded trial of an effective treatment	WLST misclassification could attenuate or amplify the observed treatment effect depending on specific trial characteristics	Unblinding may cause WLST misclassification to be lower in the treatment group (e.g., due to clinician optimism which leads to fewer withdrawals), such that more patients survive to manifest their good outcome. This exaggerates the apparent treatment benefit, even if some of that benefit is unrelated to treatment. Bias is amplified in modestly effective treatments because the true effect is small and easily inflated by this imbalance. In trials with large treatment effects, the bias may be less visible but still present
Unblinded trial of an ineffective treatment	WLST misclassification could make the treatment look beneficial	The proportion of patients with a good outcome is similar between groups. However, if the misclassification fraction is lower in the treated group (e.g., because of more cautious or delayed WLST), then more patients in the treated group will manifest their good outcome. Treatment might therefore appear effective
Unblinded trial of a harmful treatment	WLST misclassification could attenuate the perception of harm	If WLST is less likely or delayed in the treatment group due to clinician belief in its benefit, more treated patients survive to manifest good outcomes despite the treatment being harmful. This biases the observed effect toward the null, making the treatment appear less harmful

This table outlines putative mechanisms to explain the impact of WLST on trial-reported findings of patients with ABI conditions when the primary endpoint is a neurologic outcome. Other mechanisms not listed here may also be relevant. Additional mechanisms may apply to other endpoints (e.g., mortality)

## Discussion

In our literature review of 69 contemporary, high-impact trials of ABI conditions, we found that few trials reported the frequency, timing, reasons, and criteria for WLST decisions. In our simulation study, WLST misclassification led to bias in treatment effects across a wide range of trial designs, and statistical interpretations changed in a median of 22.1% of trials. These findings raise concern that many real-world trials do not provide sufficient information on WLST practices to yield confident conclusions about the true direction and magnitude of treatment effects.

### Implications of the literature review

From our literature review, most trials that reported WLST data were done in patients with cardiac arrest, reflecting the widespread recognition that WLST can introduce bias in trials of this population [4, 21, 22]. Several cardiac arrest trials used standardized protocols to guide withdrawal decisions and imposed a minimum wait period before WLST for poor neurologic prognosis could be undertaken, thereby reducing the likelihood of WLST misclassifications [23–25]. However, these considerations are also relevant in trials of other ABI populations. WLST is significantly more common in patients with ABI compared to those with non-ABI conditions [3, 11]. Early WLST mediates a significant proportion of deaths in patients with traumatic brain injury [7], ischemic stroke [26], and intracranial hemorrhage [27]. Predictions of poor neurologic outcomes—often overestimated even by experienced clinicians [28]—can create a self-fulfilling prophecy, where WLST prevents patients from receiving prolonged supportive care that might have led to meaningful recovery [29]. Although there is awareness that prognostic models for ABI may be biased by inaccurate early assessments [30], this recognition is not consistently reflected in clinical trial design. Notably, no trials in our review (except those in cardiac arrest) reported the timing, reasons, and criteria for WLST decisions. The omission of critical WLST data in contemporary ABI trials is concerning, as it raises the possibility that some patients in these studies might have been misclassified—potentially affecting trial-reported conclusions.

### Implications of the simulation study

In our simulations of trials with clinician blinding, we found that the observed treatment effect was attenuated relative to the true effect. A potential explanation is that for misclassified patients in the treatment group, WLST prevents the opportunity to respond to treatment, which leads the poor outcome probability to appear more similar between groups and reduces the observed treatment effect. One key implication of this finding is that trials may be underpowered to detect differences in outcomes if the misclassified fraction is high, because only patients whose outcome can be potentially altered by treatment contribute to the statistical power of a trial. To mitigate this risk, future trials of ABI patients (particularly those of critically ill patients receiving life-sustaining interventions) could consider incorporating a safety margin into sample size calculations that accounts for WLST misclassifications.

In contrast, in our simulations of trials where clinicians are aware of treatment allocation, as would be expected for many complex interventions, bias from WLST depended on the combination of true treatment effect strength, patients’ baseline illness severity, and misclassified fraction. In many of these scenarios, the bias from WLST was modest.

Across all simulations, approximately 22% of trials showed statistical reversal of their conclusions due to WLST-related misclassification. This proportion reflects simulation inputs and modeling choices and does not necessarily represent a universal estimate. Even where trials’ conclusions would not have been reversed by WLST, point estimates could be different—potentially compounding inaccuracies when studies are pooled in meta-analysis and influencing downstream guideline recommendations. Confident conclusions about treatment efficacy therefore require rigorous WLST reporting, including the frequency and timing of WLST events, reasons for withdrawal, and neuroprognostication criteria on which decisions were based [15]. These considerations may be less consequential in ABI trials where WLST is rare.

### Strengths and limitations

Strengths of this analysis include the use of data from published trials to create clinically relevant simulations anchored in real-world treatment effect sizes and outcome rates, the large number of simulations tested, and the focus on patient-centered outcomes. Simulations incorporated both blinded and unblinded trial scenarios, enhancing relevance to real-world trials. We used a novel quantitative framework to explore how WLST can distort treatment estimates across trial contexts, extending prior qualitative insights and highlighting generalizable patterns. This work complements a recent study that also found that WLST biases effective treatments towards the null [20]; however, our analysis explores the impact of WLST on a wider range of trial characteristics and includes exploration of neutral and harmful treatments.

This study also has some limitations. The literature screen was done by a single author and could have missed potentially eligible trials. Our search was restricted to contemporary trials published in high-impact journals, and it is possible that WLST is even less commonly reported in trials published in lower-impact journals. To improve computational efficiency, simulations used study-level features rather than patient-level data. Accordingly, our simulations should not be used to “reinterpret” the findings of published trials, but rather to explore generalizable patterns. We simulated treatment effects using the expected benefit from sample size calculations of included trials, rather than trial-reported effects, because the latter may be biased by WLST. However, expected benefits are often overly optimistic [31], which could have impacted the magnitude of bias in our simulations. We harmonized outcomes across source trials into a binary good/poor variable to provide a common construct of global neurologic outcome for the simulations; while this obscures scale-specific nuances, it captures the shared dimension most relevant to WLST-related bias. We mimicked clinician blinding and unblinding by holding the misclassified fraction constant (blinded trial simulation) or reducing it by 20 or 40% in the treated group compared to the control group (unblinded trial simulation). This approach allowed exploration of directional dynamics related to WLST imbalances, but it may also be an overly simple implementation of blinding, and does not account for different degrees of imbalance. For simplicity, we assumed that all WLST decisions led to death and did not model a scenario in which some patients survive after WLST; allowing survival would likely attenuate the bias from WLST because the misclassified fraction would be lower, and more patients’ “true” outcome would become apparent. We modeled bias arising from clinician optimism about treatment benefit, but the converse (bias arising from clinician pessimism) could also occur and would bias results in the opposite direction. We studied misclassified fractions between 2% and 20%. While a misclassified fraction of 20% may seem unlikely, proportions in this range have been reported [4, 5, 32, 33]. Furthermore, a high misclassified fraction does not imply that prognostic assessments are incorrect in 20% of cases; rather, it reflects the combined influence of real-world factors that shape withdrawal decisions beyond injury severity—including clinician experience, patient and family preferences, institutional culture, and regional practice patterns—which together introduce variability across trials [11]. Similar to other reports, we evaluated IPCW as a potential strategy to address WLST-related bias [20, 34]; however, our simplified analysis did not incorporate patient-level covariates, and further work using detailed clinical data is needed to assess the conditions under which IPCW can (and cannot) safely mitigate WLST-related bias. The timing of WLST (early vs. late) may influence bias magnitude; although not modeled explicitly, its net effect is captured within the misclassified fraction, and future patient-level simulations could examine timing more directly. In clinical practice, WLST decisions evolve dynamically, but in our simulations, they were modeled as post-randomization events dependent on patients’ true outcomes—recognizing that this is a simplification of a complex causal process. We did not study the impact of WLST on mortality because a good neurologic outcome is arguably a more patient-centered endpoint in ABI trials. Finally, the proportion of trials in which WLST reversed findings reflects the specific simulation parameters and modeling assumptions and should not be interpreted as a universal estimate.

## Conclusions

In conclusion, we found that few high-impact, contemporary ABI trials report data on WLST events or timing, reasons, and criteria for withdrawal decisions. WLST misclassification may distort treatment effects in clinical trials that measure neurologic outcomes, precluding confident conclusions about treatment efficacy. Future trials in ABI patients should standardize WLST decisions—an effort that may also improve clinical prognostication—and report WLST data to support accurate interpretation of their findings.

## Supplementary Information

Supplementary Material 1

## Data Availability

Statistical code used in this study is available from the corresponding author upon reasonable written request.

## Abbreviations

ABI: acute brain injury
IPCW: inverse probability censoring weighting
IQR: interquartile range
WLST: withdrawal of life-sustaining treatment
