# Supplementary material for: Reporting practices and impact of withdrawal of life-sustaining treatment on outcomes in acute brain injury clinical trials: a literature review and simulation study
Source: Crit Care. 2026 Mar 8;30:181. doi: 10.1186/s13054-026-05929-7 (PMC13081334; doi:10.1186/s13054-026-05929-7)
Supplement: Supplementary file 1 — Supplementary Material 1 [file 13054_2026_5929_MOESM1_ESM.docx]

**Online supplement**

Shaurya Taran, MD; Jeffrey M. Singh, MD MSc; Christopher J. Yarnell, MD PhD; Victoria A. McCredie, MBChB PhD; Damon C. Scales, MD PhD; Niall D. Ferguson, MD MSc; Kuan Liu, PhD; Neill K.J. Adhikari, MDCM MSc

**eSupplement1: Search Strategy, Journal Selection, and Screening**

We searched Pubmed from January 1, 2015 through December 19, 2024 using the following search terms:

((Intracranial Hemorrhage[MeSH Terms] OR Subarachnoid Hemorrhage[MeSH Terms] OR Traumatic Brain Injury[MeSH Terms] OR Cardiac Arrest[MeSH Terms] OR Ischemic Stroke[Title/Abstract] OR "severe brain injury"[Title/Abstract] OR "acute brain injury"[Title/Abstract])

AND (randomized controlled trial[Publication Type] OR randomized[Title/Abstract] OR randomised[Title/Abstract])

AND (Lancet Neurol[Journal] OR JAMA[Journal] OR JAMA Neurol[Journal] OR Lancet[Journal] OR Intensive Care Med[Journal] OR Lancet Respir Med[Journal] OR N Engl J Med[Journal] OR BMJ[Journal] OR Neurology[Journal] OR Am J Respir Crit Care Med [Journal])

AND ("2015/01/01"[Date - Publication]: "2024/12/19"[Date - Publication]))

Journal selection was based on consensus among the interdisciplinary authorship team, which included intensivists, neuro-intensivists, statisticians, and clinical trialists with experience conducting trials in patients with acute brain injuries (ABI). The primary selection criterion was journals that were likely to publish practice-changing clinical trials relevant to the care of patients with ABI. All articles were screened by title and abstract by a single author (ST) using Endnote 2025. Articles meeting inclusion criteria were reviewed in full text by two authors (ST, NKJA), and a random audit of 100 excluded articles was performed by the supervising author (NKJA).

We excluded trials of patients on extracorporeal life support, feasibility trials, non-inferiority trials, trials with factorial designs, trials of non-clinical primary outcomes, trials with more than two study arms, and trials in non-human subjects. For stroke trials, we excluded any studies that evaluated antiplatelet or thrombolytic therapies in isolation of other interventions. The rationale was that illness severity of most patients in these trials was low and withdrawal of life sustaining-treatment (WLST) may be less relevant to the interpretation of their findings.

Data from each trial was extracted into a standardized case report form. The following trial-level data were collected: name of the trial, journal and year of publication, condition studied, details of the intervention, sample size, primary outcome, expected effect size, and key findings. The following information related to WLST was also collected: frequency, timing of events, reasons for WLST, and whether neuroprognostication before WLST was standardized. Data extraction was done by a single author (ST) and a random audit of completed case report forms was done by the senior author (NKJA). All information was collected from the primary article and associated supplementary materials. We did not contact study authors in case WLST information was not reported in these sources.

**eSupplement2: Additional methods**

This section provides additional aspects regarding the simulation study of hypothetical clinical trials. Details are presented following the recommendations of Morris et al.^1^

**Aims:** We aimed to evaluate the impact of withdrawal of life-sustaining treatment (WLST) misclassification on intervention effect sizes reported in randomized clinical trials. Specifically, we examined the difference between the true treatment effect (i.e., the effect intrinsic to the intervention under study) and the observed treatment effect (i.e., the result that would be reported in a clinical trial after WLST misclassification occurs). Simulations were performed for a range of different treatment effect sizes, trial sample sizes, and WLST misclassified fractions. All simulated trials measured a neurologic outcome (binary or ordinal), as described in the main manuscript.

**Estimands:** Two estimands were derived in each simulation, including the *observed treatment effect* and the *bias* (defined as the difference between true and observed treatment effects). Larger differences in the bias parameter suggest a greater impact of WLST misclassifications on outcomes reported in a clinical trial.

**Methods:** We varied the following inputs across simulations: true treatment effect, baseline probability of a poor outcome in the control group, sample size, and WLST misclassified fraction. All simulations used trial-level inputs from the literature review; we did not use patient-level covariates in any analyses. Furthermore, in our framework, WLST probability was conditioned on true outcome status, simplifying heterogeneity while preserving the conceptual link between prognosis and withdrawal risk. The following section describes how each input was selected and combined across trials.

*True treatment effect*: In our literature review, the expected treatment effect size (expressed in terms of absolute risk reduction for a poor outcome) across trials with a binary primary outcome ranged from 1.3% in a trial of continuous cardiopulmonary resuscitation for out-of-hospital cardiac arrest,^2^ to 30% in a trial of general anesthesia versus conscious sedation in stroke.^3^ We selected five different treatment effects for simulation using quintiles of the absolute risk reduction; these values corresponded approximately to 1.5%, 6%, 10%, 14%, and 17%. In addition, because many treatments investigated in randomized trials are ineffective and some may be harmful, we added two effect sizes of 0% (i.e., there is no difference between the treated and untreated) and -5% (i.e., there is an absolute *increase* in poor outcomes by 5% among the treated). For our simulations, we considered the true treatment effect to be equivalent to the absolute risk reduction in poor neurologic outcomes in the treated group, compared to the control group, in a clinical trial where no WLST misclassification occurs.

*Baseline probability of a poor outcome in the control group:* We used data from control group outcomes across 43 trials that reported a binary primary outcome. Probabilities of a baseline poor outcome ranged from 5.4% in a trial of patent foramen ovale closure^4^ to 97.6% in a trial of epinephrine for out-of-hospital cardiac arrest.^5^ We selected poor outcome probabilities using quintiles of control-group outcomes across trials in our literature review; this gave values of approximately 6%, 37%, 50%, 63%, and 86%. Importantly, “poor outcome” was variably defined across treatment conditions and trials and could have represented, for example, Cerebral Performance Category scores of 3-5 (denoting severe disability, coma/vegetative state, or death) in a trial of trans-nasal cooling following out-of-hospital cardiac arrest,^6^ modified Rankin Scores of 4-6 (denoting moderately severe disability, severe disability, or death) in a trial of thrombectomy in ischemic stroke,^7^ or mortality in a trial examining tranexamic acid in moderate to severe traumatic brain injury.^8^ The timing of outcome ascertainment was also variable across trials. Different poor outcome probabilities between trials may therefore simply reflect differences in the study population, the scale used to measure outcomes, or the timing of follow-up. For our simulations, we used study-level definitions of the poor-outcome probability in order to make use of all available data across all clinical conditions and follow-up durations.

*Sample size*: We derived sample sizes for each simulated trial according to the expected absolute risk reduction and poor outcome probability in the control group. For all sample size calculations, we assumed an alpha level of 0.05 and a beta level of 0.20. In simulations where the true ARR was 0% or -5%, the sample size was calculated assuming an expected ARR of 7% in poor outcomes among the treated, compared to the untreated (i.e., the sample size was selected with the assumption that treatment would show a small benefit, otherwise there would be no equipoise to conduct the trial).

*WLST misclassified fraction*: Some patients who might experience a good neurologic outcome could receive WLST (this is the expected mechanism that contributes to bias in a clinical trial). We refer to the proportion of WLST events that occur in such patients as the *misclassified fraction*. To capture this phenomenon, we fixed the misclassified fraction at 2%, 5%, 10%, and 20% in different simulations. The upper bound (20%) was chosen based on observational data which suggests that 16-19% of patients with cardiac arrest who undergo WLST could have achieved functionally favourable outcomes,^9,10^ and up to 30% of patients with traumatic brain injury could have achieved partial independence.^11^ We chose a more conservative upper limit of 20% for our simulations under the assumption that WLST would be applied more rigorously in randomized trials than in unstructured real-world settings. The 2%, 5%, and 10% quantities were chosen based on group consensus. Notably, the misclassified fraction includes the impact of incorrect prognostic assessments, which could have led to WLST in patients who would have otherwise experienced a good neurologic outcome, in addition to the influence of other factors (e.g., institutional culture, resource availability, patient or substitute decision-maker wishes) not fully related to ABI severity.

*Combining inputs:* We matched each of the seven ARRs to each of the five baseline probabilities of a poor outcome. We then generated sample sizes for all resulting pairs. We discarded any simulations where the calculated sample size exceeded the largest trial from our literature review, on the basis that such a trial would be unlikely to occur. This led to removal of the 37% and 50% baseline poor outcome probability scenarios for trials with an ARR of 1.5%, because these would have required sample sizes of 32,244 and 34,874 patients, respectively (i.e., sample sizes would have been larger than the largest trial identified in our literature review). We also discarded simulations where the specified ARR exceeded the baseline probability of a poor outcome in the control group (e.g., ARR of 10% when the expected baseline probability of a poor outcome is 6%). Finally, we paired each remaining set of ARR/outcome groupings to the four misclassified fractions.

**Data generating mechanisms:** All simulations were designed to mimic two-arm superiority randomized clinical trials. We randomized patients into the intervention or control group with a 50:50 probability using resampling with replacement from a Bernoulli distribution. In the main analysis, the study outcome was a binary variable (poor outcome = death or poor neurologic outcome; good outcome = survival with a good neurologic outcome, including up to moderate disability). We assigned patients to their “true” outcome using random draws from a binomial distribution. The proportion of patients with a poor outcome in the control group was defined according to our literature review (i.e, 37%, 50%, 63%, or 86%). The proportion of patients with a poor outcome in the treatment group was derived as the difference between the control group outcome and the true treatment effect. Next, we simulated the impact of WLST misclassification on clinical trial outcomes. In each simulation, we used random draws from a binomial distribution to assign WLST status based on underlying prognosis. Two different analyses were done. First, we fixed the misclassified fraction at the same value between the treated and control groups (i.e., patients had an equal probability of being misclassified, as might occur in a blinded trial). Second, we decreased the misclassified fraction by 20% in the treated group, compared to the control (i.e., patients in the treated group had a slightly lower probability of being misclassified, potentially reflecting a more conservative WLST strategy among the treated, as might occur in an unblinded trial). For the analysis of a neutral treatment (i.e., ARR=0%) in the unblinded trial scenario, we also studied a misclassified fraction of 40% lower in the treated, reflecting a strong belief in efficacy that leads to even fewer WLST events in those exposed to the intervention. The 20% and 40% quantities were chosen based on group consensus.

In a secondary analysis, we studied the impact of WLST on an ordinal outcome. For this analysis, we used control group outcome probabilities from two published trials. The first was a blood transfusion trial in patients with heterogeneous ABI conditions in which the outcome was defined using the eight-level Glasgow Outcome Score extended (GOSe), where 1=death (worst possible outcome) and 8=upper-good recovery (best possible outcome).^12^ The second was a thrombectomy trial in patients with basilar stroke in which the outcome was defined using the seven-level modified Rankin Scale (mRS), where 0=no symptoms (best possible outcome) and 6=death (worse possible outcome).^13^ We combined scores of 0 and 1 in this trial because only 1% of patients had a score of 0 in the control group. For the simulations, we used control group outcomes (as reported in the trials) from an eight and six-level categorical distribution, respectively. We generated treatment group probabilities using the trial-reported proportional odds ratios. Corresponding confidence intervals were derived using the sample size of each trial (these values may be slightly different from the published trials because of simulation-related variability). We then applied WLST misclassification using random draws from a Bernoulli distribution. We studied four different misclassified fractions as previously described.

**Repetitions and Outputs:** We ran 1,000 iterations of each simulation and reported model outputs as the mean of each quantity of interest along with 95% confidence intervals, derived non-parametrically from the 2.5^th^ and 97.5^th^ percentile. Confidence intervals represent the distribution of point estimates of the ARRs from 1,000 trials each using the same inputs. Confidence intervals for the observed treatment effect and bias in the following tables in this appendix are based on these values (not reported in the main paper). We also reported Monte Carlo errors to capture variability around the mean effect arising from the simulation process. These confidence intervals are reported in Figures 2 and 3 in the main paper and also in the following tables in the appendix.

**Clinical assumptions:** We made the following additional assumptions: 1) all WLST events occurred after patients were randomized, 2) all WLST events occurred before outcome ascertainment, 3) all WLST events led to death, 4) decisions to perform WLST were independent across patients (i.e. the decision to withdraw treatment in one patient did not influence the decision to withdraw treatment in another patient), 5) randomization resulted in fully exchangeable groups at baseline, 6) all patients received the treatment to which they were assigned (i.e., full adherence to the study protocol with no crossovers), and 7) complete follow-up information was available for all randomized patients.

**Evaluating a statistical approach to attenuate bias:** In an additional exploratory analysis, we used inverse probability of censoring weighting (IPCW) to assess whether bias from WLST-related misclassification could be attenuated. We applied unstabilized IPCW by calculating censoring weights as the inverse of the probability of remaining uncensored, conditional on the true outcome. We did not trim the weights. Weights were then used to up-weight non-censored observations, creating a pseudo-population that approximates the distribution of outcomes had no censoring occurred. Finally, we calculated the treatment effect as the absolute risk reduction in poor outcome probability between groups in this pseudo-population and compared it to the “known” true treatment effect. This simplified IPCW analysis assumed censoring depended only on outcome and excluded patient-level covariates. Unmeasured confounding likely persists, and the approach requires further validation in future studies. Accordingly, this exercise illustrates IPCW’s conceptual behavior under idealized conditions rather than its adequacy for real trial data.

**eTable1: Trials with a binary primary outcome**

| **Trial** | **Journal**  **(Year)** | **Condition** | **Intervention** | **Sample size (n)** | **Primary outcome** | **Expected effect size** | **Key findings** | **WLST (%)** |
| --- | --- | --- | --- | --- | --- | --- | --- | --- |
|  |  |  |  |  |  |  |  |  |
| TTM2 | NEJM  (2021) | Cardiac arrest | Hypothermia | 1,900 | 6-month mortality | 7.5% absolute reduction in mortality | Treatment group: 50% mortality  Control group: 48% mortality | NR ^a^ |
| CRASH-3 | Lancet (2019) | Traumatic brain injury | Tranexamic acid | 12,737 | 28-day mortality | 3% absolute reduction in mortality | Treatment group: 18.5% mortality  Control group: 19.8% mortality | NR |
| OPTIMAL-BP | JAMA  (2023) | Ischemic stroke | Blood pressure control | 306 | 3-month mRS | 11% absolute reduction in dependence or death | Treatment group: 60.6% mRS 3-6  Control group: 45.6% mRS 3-6 | NR |
| RESCUE-Japan-LIMIT | NEJM (2022) | Ischemic stroke | Thrombectomy | 203 | 90-day mRS | 15.3% absolute reduction in unfavourable outcomes | Treatment group: 69.0% mRS 4-6  Control group: 87.3% mRS 4-6 | NR |
| EARLYDRAIN | JAMA Neurology (2023) | Subarachnoid hemorrhage | Lumbar drain | 287 | 6-month mRS | 17% absolute reduction in unfavourable outcome | Treatment group: 32.6% mRS 3-6  Control group: 44.8% mRS 3-6 | NR |
| PARAMEDIC2 | NEJM (2018) | Cardiac arrest | Epinephrine | 8,014 | 30-day mortality | 1.5% absolute reduction in 30-day mortality | Treatment group: 96.8% mortality  Control group: 97.6% mortality | NR |
| TAME | NEJM (2023) | Cardiac arrest | Hypercapnia | 1,700 | 6-month GOSe | 8% absolute reduction in poor neurologic outcome | Treatment group: 56.6% GOSe 1-4  Control group: 55.4% GOSe 1-4 | 29.3% ^b^ |
| MISTIE III | Lancet (2019) | Intracranial hemorrhage | Hematoma evacuation | 506 | 365-day mRS | 13% absolute reduction in unfavourable outcome | Treatment group: 55% mRS 4-6  Control group: 59% mRS 4-6 | 12.2% |
| HYPERION | NEJM (2019) | Cardiac arrest | Hypothermia | 584 | 90-day CPC | 9% absolute reduction in unfavourable neurologic outcome | Treatment group: 89.8% CPC 3-5  Control group: 94.3% CPC 3-5 | 63.5 |
| Prehospital TXA for TBI trial | JAMA  (2020) | Traumatic brain injury | Tranexamic acid | 1063 | 6-month GOSe | 10% absolute reduction in unfavourable neurologic outcome | Treatment group: 35.0% GOSe 1-4  Control group: 38.0% GOSe 1-4 | NR |
| TRAIN | JAMA (2024) | Acute brain injury | Blood transfusion | 850 | 180-day GOSe | 5% absolute reduction in unfavourable outcome | Treatment group: 62.6% GOSe 1-5  Control group: 72.6% GOSe 1-5 | NR |
| ATACH-2 | NEJM (2016) | Intracranial hemorrhage | Blood pressure control | 1,000 | 3-month mRS | 10% absolute reduction in death or disability | Treatment group: 38.7% mRS 4-6  Control group: 37.7% mRS 4-6 | 4.6% |
| TELSTAR | NEJM (2022) | Cardiac arrest | Antiseizure medications | 172 | 3-month CPC | 7% absolute reduction in poor outcome | Treatment group: 90% CPC 3-5  Control group: 92% CPC 3-5 | 77.8% |
| ULTRA | Lancet (2021) | Subarachnoid hemorrhage | Tranexamic acid | 955 | 6-month mRS | 8.1% absolute reduction in poor outcome | Treatment group: 40% mRS 4-6  Control group: 36% mRS 4-6 | NR |
| ANTHARTIC | NEJM (2019) | Cardiac arrest | Amoxicillin-clavulanate | 198 | 7-day VAP | 25% absolute reduction in VAP | Treatment group: 19% VAP  Control group: 34% VAP | 29.3% ^c^ |
| AIRWAYS-2 | JAMA (2018) | Cardiac arrest | Airway management | 9,296 | mRS at hospital discharge or 30 days | 2% absolute reduction in poor outcome | Treatment group: 93.6% mRS 4-6  Control group: 93.2% mRS 4-6 | NR |
| BEST | Lancet Neurology (2020) | Ischemic stroke | Thrombectomy | 131 | 90-day mRS | 15% absolute reduction in poor outcome | Treatment group: 58% mRS 4-6  Control group: 68% mRS 4-6 | NR |
| PARAMEDIC | Lancet (2015) | Cardiac arrest | LUCAS device | 4,471 | 30-day mortality | 2.5% absolute reduction in mortality | Treatment group: 94% mortality  Control group: 93% mortality | NR |
| EXACT | JAMA (2022) | Cardiac arrest | Oxygen | 428 | Hospital mortality | 9% absolute reduction in mortality | Treatment group: 61.7% mortality  Control group: 52.1% mortality | 51.3% |
| POLAR | JAMA (2018) | Traumatic brain injury | Hypothermia | 511 | 6-month GOSe | 15% absolute reduction in unfavourable outcome | Treatment group: 51.2% GOSe 1-4  Control group: 50.9% GOSe 1-4 | NR |
| SWITCH | Lancet (2024) | Intracranial hemorrhage | Decompressive craniectomy | 201 | 180-day mRS | 17.5% absolute reduction in unfavourable outcome | Treatment group: 44% mRS 5-6  Control group: 58% mRS 5-6 | NR |
| ARREST | Lancet (2023) | Cardiac arrest | Hospital transfer | 862 | 30-day mortality | 10% absolute reduction in mortality | Treatment group: 63% mortality  Control group: 63% mortality | NR |
| ROC | NEJM (2015) | Cardiac arrest | Continuous CPR | 23,711 | Hospital mortality | 1.3% absolute reduction in mortality | Treatment group: 91% mortality  Control group: 90.3% mortality | NR |
| PRINCESS | JAMA (2019) | Cardiac arrest | Trans-nasal cooling | 677 | 90-day CPC | 16% absolute reduction in poor neurologic outcome | Treatment group: 83.4% CPC 3-5  Control group: 86.5% CPC 3-5 | NR |
| Kirkegaard et al. | JAMA (2017) | Cardiac arrest | Hypothermia | 355 | 6-month CPC | 15% absolute difference in poor neurologic outcome | Treatment group: 31% CPC 3-5  Control group: 36% CPC 3-5 | 15.9% |
| HEMOTION | NEJM (2024) | Traumatic brain injury | Blood transfusion | 742 | 6-month GOSe | 10% absolute reduction in unfavourable outcome | Treatment group: 68.4% unfavourable outcome ^d^  Control group: 73.5% unfavourable outcome | 17.0% |
| CHOICE | JAMA (2022) | Ischemic stroke | Intra-arterial therapy | 121 | 90-day mRS | 19% absolute reduction in poor neurologic outcomes | Treatment group: 41% mRS 2-6  Control group: 59.6% mRS 2-6 | NR |
| CMOSS | JAMA (2023) | Ischemic stroke | EC-IC bypass | 324 | Stroke or death in 30 days | 14% absolute reduction in stroke or death | Treatment group: 8.6% stroke or death  Control group: 12.3% stroke or death | NR |
| TOMAHAWK | NEJM (2021) | Cardiac arrest | Percutaneous coronary intervention | 554 | 30-day mortality | 12% absolute reduction in mortality | Treatment group: 54% mortality  Control group: 46% mortality | 11.1% |
| SHINE | JAMA (2019) | Ischemic stroke | Insulin | 1,151 | 90-day mRS | 7% absolute reduction in unfavourable outcomes ^e^ | Treatment group: 79.5% unfavourable outcome  Control group: 78.4% unfavourable outcome | 2.5% |
| BASIS | JAMA (2024) | Ischemic stroke | Balloon angioplasty | 512 | Stroke or death in 30 days | 8% absolute reduction in stroke or death | Treatment group: 4.4% stroke or death  Control group: 13.5% stroke or death | NR |
| PROPHY-VAP | Lancet Resp Med (2024) | Acute brain injury | Ceftriaxone | 345 | 7-day VAP | 15% absolute reduction in VAP | Treatment group: 14.2% VAP  Control group: 32.5% VAP | NR |
| PEACH | Lancet Neurology (2022) | Intracranial hemorrhage | Antiseizure medication | 50 | Seizure within 72 hrs | 25% absolute reduction in seizures | Treatment group: 15.8% seizures  Control group: 43.5% seizures | NR |
| AMETIS | JAMA Neurology (2023) | Ischemic stroke | General anesthesia | 273 | 90-day functional independence ^f^ | 20% absolute reduction in unfavourable outcome | Treatment group: 71.8% unfavourable outcome  Control group: 63.8% unfavourable outcome | NR |
| CANVAS II | JAMA Neurology (2022) | Ischemic stroke | Conscious sedation | 87 | 90-day mRS | 30% absolute reduction in unfavourable outcome | Treatment group: 45.5% mRS 3-6  Control group: 51.2% mRS 3-6 | NR |
| VICTOR | BMJ  (2024) | Cardiac arrest | Intraosseous access | 1,771 | Survival to hospital discharge | 5% absolute reduction in mortality | Treatment group: 89.3% mortality  Control group: 89.7% mortality | NR |
| PROLABI | AJRCCM (2024) | Acute brain injury | Lung protective ventilation | 190 | Vent dependence, death or ARDS | 11% absolute reduction in composite event rate | Treatment group: 61.5% composite event  Control group: 45.7% composite event rate | NR^g^ |
| VISSIT | JAMA (2015) | Ischemic stroke | Balloon-expandable stent | 112 | Stroke or TIA within 12 months | 20% absolute reduction in stroke or TIA | Treatment group: 36.2% stroke or TIA  Control group: 15.1% stroke or TIA | NR |
| COACT | NEJM (2019) | Cardiac arrest | Percutaneous coronary intervention | 552 | 90-day mortality | 13% absolute reduction in mortality | Treatment group: 35.5% mortality  Control group: 32.8% mortality | 26.3% |
| OXY-TC | Lancet Neurology (2023) | Traumatic brain injury | Brain tissue oxygen monitoring | 318 | 6-month GOSe | 17% absolute reduction in unfavourable outcomes | Treatment group: 53.0% GOSe 1-4  Control group: 51.8% GOSe 1-4 | 3.4% |
| BAOCHE | NEJM (2022) | Ischemic stroke | Thrombectomy | 217 | 90-day mRS | 20% absolute reduction in unfavourable outcomes | Treatment group: 53.6% mRS 4-6  Control group: 75.7% mRS 4-6 | NR |
| ATTENTION | NEJM (2022) | Ischemic stroke | Thrombectomy | 340 | 90-day mRS | 20% absolute reduction in unfavourable outcomes | Treatment group: 54.0% mRS 4-6  Control group: 77.2% mRS 4-6 | NR |
| CAPITAL CHILL | JAMA (2021) | Cardiac arrest | Hypothermia | 367 | 180-day DRS | 15% absolute reduction in poor outcome | Treatment group: 48.4% DRS >5  Control group: 45.4% DRS >5 | 34.3% |
| Gore REDUCE | NEJM (2017) | Ischemic stroke | PFO closure | 664 | 24-month recurrence of stroke ^h^ | 4.4% absolute reduction in stroke recurrence | Treatment group: 1.4% stroke  Control group: 5.4% stroke | NR |
| Wang et al. | JAMA (2018) | Cardiac arrest | Airway management | 3,004 | 72-hour mortality | 4.5% absolute reduction in mortality | Treatment group: 81.7% mortality  Control group: 84.6% mortality | NR |
| ACES | ICM (2023) | Traumatic brain injury | Median nerve stimulation | 329 | Recovery of consciousness at 6 months | 13.6% absolute reduction in patients remaining comatose | Treatment group: 27.5% comatose  Control group: 43.2% comatose | NR |

*Table legend:*

^a^ Reported in the supplement in a histogram but raw numbers were not included. Values in the histogram for this particular trial could not be reliably added.

^b^ Derived as an approximation by adding histogram bars in supplement.

^c^ Reported as neurologic/care limitation and defined between groups.

^d^ Used a sliding dichotomy where outcome was based on baseline prognosis.

^e^ A favorable outcome was defined as a modified Rankin Scale score of 0 in patients with a baseline NIHSS score of 3 to 7, a modified Rankin Scale score of 0 to 1 in patients with a baseline NIHSS score of 8 to 14, and a modified Rankin Scale score of 0 to 2 in patients with a baseline NIHSS score of 15 to 22.

^f^ The prespecified primary composite outcome was functional independence (a score of 0 to 2 on the modified Rankin Scale, which ranges from 0 [no neurologic disability] to 6 [death]) at 90 days and absence of major periprocedural complications (procedure-related serious adverse events, pneumonia, myocardial infarction, cardiogenic acute pulmonary edema, or malignant stroke) at 7 days.

^g^ The authors report the following: “Reasons for death were recorded, and no indications for withdrawal of life-sustaining therapy were applied in patients enrolled in the study.” It is not clear if the authors meant that WLST was prohibited or simply left to the discretion of treating clinicians without standardized criteria. An NR value was assigned to this particular trial with the rationale that clear reporting is necessary for appropriate interpretation of trial outcomes.

^h^ The coprimary end points were freedom from clinical evidence of ischemic stroke (reported here as the percentage of patients who had a recurrence of stroke) through at least 24 months after randomization and the 24-month incidence of new brain infarction, which was a composite of clinical ischemic stroke or silent brain infarction detected on imaging.

*Abbreviations:* ARDS, acute respiratory distress syndrome; CPC, cerebral performance category; CPR; cardiopulmonary resuscitation; DRS, disability rating scale; EC-IC; extracranial-intracranial; GOSe, Glasgow outcome score extended; mRS, modified Rankin Scale; NR, not reported; PFO, patent foramen ovale; TIA, transient ischemic attack; VAP, ventilator associated pneumonia; WLST, withdrawal of life-sustaining treatment

|  |  |  |  |  |  |  |  |  |
| --- | --- | --- | --- | --- | --- | --- | --- | --- |
|  |  |  |  |  |  |  |  |  |

**eTable2: Trials with an ordinal primary outcome**

| **Trial** | **Journal**  **(Year)** | **Condition** | **Intervention** | **Sample size** | **Primary outcome** | **Expected effect size** | **Key findings** | **WLST** |
| --- | --- | --- | --- | --- | --- | --- | --- | --- |
|  |  |  |  |  |  |  |  |  |
| ENRICH | NEJM (2024) | Intracranial hemorrhage | Hematoma evacuation | 300 | 180-day mRS | 15% absolute reduction in poor neurologic outcome | Hematoma evacuation superior to control  Control group: 82% mRS 4-6 | NR |
| MR CLEAN | NEJM (2015) | Ischemic stroke | Intra-arterial therapy | 500 | 90-day mRS | 10% absolute reduction in poor neurologic outcomes | Thrombectomy superior to control  Control group: 64% mRS 4-6 | NR |
| MR CLEAN LATE | Lancet (2023) | Ischemic stroke | Thrombectomy | 535 | 90-day mRS | Not reported on absolute scale | Thrombectomy superior to control  Control group: 58% mRS 4-6 | NR |
| TICH 2 | Lancet (2018) | Intracranial hemorrhage | Tranexamic acid | 2,325 | 90-day mRS | Not reported on absolute scale | No difference in 90-day mRS  Control group: 54.7% mRS 4-6 | NR |
| ENCHANTED | Lancet (2019) | Ischemic stroke | Blood pressure control | 2,196 | 90-day mRS | 7% absolute reduction in poor neurologic outcome | No difference in 90-day mRS  Control group: 22.7% mRS 4-6 | 2.6% |
| GRECO | JAMA Neurology | Cardiac arrest | Ghrelin | 160 | 6-month CPC | Not reported on absolute scale | Ghrelin potentially superior to control  Control group: 58% CPC 4-5 | 30% |
| RESILIENT | NEJM (2020) | Ischemic stroke | Thrombectomy | 300 | 90-day mRS | Not reported on absolute scale | Thrombectomy superior to control  Control group: 65% mRS 4-6 | NR |
| FAST-MAG | NEJM (2015) | Stroke | Magnesium sulphate | 1,700 | 90-day mRS | Not reported on absolute scale | No difference in functional outcomes  Control group: 33.8% mRS 4-6 | NR |
| MR ASAP | Lancet Neurology (2022) | Stroke (ischemic or hemorrhagic) | Nitroglycerin | 325 | 90-day mRS | 7% absolute reduction in poor neurologic outcome | No difference in functional outcomes  Control group: 31% mRS 4-6 | NR |
| DAWN | NEJM (2017) | Ischemic stroke | Thrombectomy | 206 | 90-day mRS | NA (Bayesian design) | Thrombectomy superior to control  Control group: 70% mRS 4-6 | NR |
| DEFUSE 3 | NEJM (2018) | Ischemic stroke | Thrombectomy | 182 | 90-day mRS | NA (Adaptive enrichment design) | Thrombectomy superior to usual care  Control group: 69% mRS 4-6 | NR |
| CHAIN | Lancet (2024) | Intracranial hemorrhage | Traditional Chinese medicine | 1,648 | 90-day mRS | 6% absolute reduction in poor neurologic outcome | No difference in 90-day mRS  Control group: 38.1% mRS 4-6 | NR |
| LASTE | NEJM (2024) | Ischemic stroke | Thrombectomy | 333 | 90-day mRS | 1-point improvement in mRS with common OR of 1.65 | Thrombectomy superior to control  Control group: 87.3% mRS 4-6 | 0.3% |
| COBI | JAMA (2021) | Traumatic brain injury | Hypertonic saline | 370 | 6-month GOSe | 14% absolute reduction in poor neurologic outcome | No difference in 6-month GOSe  Control group: 50% GOSe 1-4 | NR |
| MARVEL | JAMA (2024) | Ischemic stroke | Dexamethasone | 1,680 | 90-day mRS | 7% absolute reduction in poor outcomes | No difference in 90-day mRS  Control group: 45.3% mRS 4-6 | NR |
| REVASCAT | NEJM (2015) | Ischemic stroke | Thrombectomy | 206 | 90-day mRS | Not reported on absolute scale | Thrombectomy superior to control  Control group: 52.4% mRS 4-6 | NR |
| RACECAT | JAMA (2022) | Ischemic stroke | Pre-hospital transport | 1,401 | 90-day mRS | 6% absolute reduction in poor outcome | No difference in 90-day mRS  Control group: 47.7% mRS 4-6 | NR |
| SWIFT PRIME | NEJM (2015) | Ischemic stroke | Thrombectomy | 196 | 90-day mRS | Not reported on absolute scale | Thrombectomy superior to control  Control group: 69% mRS 4-6 | NR |
| HeadPoST | NEJM (2017) | Ischemic stroke | Head positioning | 11,093 | 90-day mRS | Not reported on absolute scale | No difference in 90-day mRS  Sitting up group: 23.4% mRS 4-6 | NR |
| RESCUE-ASDH | NEJM (2023) | Traumatic brain injury | Hematoma evacuation | 228 | 12-month GOSe | 8% absolute reduction in poor neurologic outcome | No difference in 12-month GOSe  Craniotomy group: 63.2% GOSe 1-4 | NR |
| SELECT-2 | NEJM (2023) | Ischemic stroke | Thrombectomy | 352 | 90-day mRS | Not reported on absolute scale | 90-day mRS lower in treatment group  Control group: 79.9% mRS 4-6 | NR |
| TENSION | Lancet (2023) | Ischemic stroke | Thrombectomy | 253 | 90-day mRS | Not reported on absolute scale | Thrombectomy superior to control  Control group: 86.9% mRS 4-6 | NR |
| RESCUEicp | NEJM (2016) | Traumatic brain injury | Decompressive craniectomy | 408 | 6-month GOSe | 15% absolute reduction in poor outcome | Craniectomy led to more survivors with significant disability  Control group: 70.4% GOSe 1-4 | NR |

*Abbreviations:* CPC, cerebral performance category; GOSe, Glasgow outcome score extended; mRS, modified Rankin scale; NA, not applicable; NR, not reported; WLST, withdrawal of life-sustaining treatment

**eTable3: Additional details of trials reporting WLST frequency**

| **Trial** | **Condition** | **Did trial report timing of WLST events?** | **Did trial report reasons for WLST?** | **Was neuroprognostication standardized?** |
| --- | --- | --- | --- | --- |
|  |  |  |  |  |
| TTM2 | Cardiac arrest | Yes | Yes | Yes |
| TAME | Cardiac arrest | Yes | Yes | Yes |
| MISTIE III | Intracranial hemorrhage | No | No | No ^a^ |
| HYPERION | Cardiac arrest | Yes | Yes | Yes |
| ATACH 2 | Intracranial hemorrhage | No | No | Unknown ^b^ |
| TELSTAR | Cardiac arrest | Yes | No | Yes |
| ANTHARCTIC | Cardiac arrest | No | Yes | Unknown ^b^ |
| CAPITAL CHILL | Cardiac arrest | Yes | Yes | Yes |
| EXACT | Cardiac arrest | Yes | Yes | Unknown ^b^ |
| Kirkegaard et al. | Cardiac arrest | Yes ^c^ | Yes | Yes |
| HEMOTION | Traumatic brain injury | No | No | No |
| TOMAHAWK | Cardiac arrest | No | Yes ^d^ | No ^e^ |
| SHINE | Ischemic stroke | No | No | No |
| COACT | Cardiac arrest | Yes | Yes | No ^f^ |
| OXY-TC | Traumatic brain injury | No | No | No |
| ENCHANTED | Ischemic stroke | No | No | No |
| GRECO | Cardiac arrest | Yes | Yes | Yes |
|  |  |  |  |  |

*Table legend:*

^a^ From the study supplement: Discussions of prognosis and decisions to continue, limit, or withdraw life-sustaining interventions will be conducted according to each institution’s policies for end-of-life decision-making, as well as their institutional codes of medical ethics.

^b^ Details were not given in the main study or supplement.

^c^ Data was reported as time to death in patients who had WLST.

^d^ Data was reported as causes of death, which also included deaths from WLST.

^e^ From the study supplement: Withdrawal of life-sustaining therapies was left to the discretion of the treating physicians.

^f^ From the study: The approach to withdrawal of life-sustaining treatment for patients with persistent coma was not prespecified and was based on local practice

*Abbreviations:* WLST, withdrawal of life-sustaining treatment

**eTable4: Bias from WLST when treatment leads to an ARR of 1.5% in poor outcomes**

| **Misclassified fraction** | **Control group poor outcome probability (%)** | **Sample size (n)** | **Observed ARR after WLST, (95% CI)** | **Bias, (95% CI)** | **MCSE** | **% trials with reversal of statistical interpretation** |
| --- | --- | --- | --- | --- | --- | --- |
|  |  |  |  |  |  |  |
| 2% | 6 | 6,938 | 1.48  (0.36, 2.70) | -0.02  (-1.14, 1.20) | 1.47  (1.46,1.48) | 32.1 |
|  | 86 | 17,544 | 1.47  (0.45, 2.52) | -0.03  (-1.05, 1.02) | 1.48  (1.46, 1.49) | 22.2 |
| 5% | 6 | 6,938 | 1.42  (-0.04, 2.85) | -0.08  (-1.54, 1.35) | 1.42  (1.41, 1.44) | 46.7 |
|  | 86 | 17,544 | 1.40  (0.34, 2.44) | -0.10  (-1.16, 0.94) | 1.42  (1.41, 1.43) | 23.6 |
| 10% | 6 | 6,938 | 1.30  (-0.36, 3.07) | -0.20  (-1.86, 1.57) | 1.36  (1.35, 1.38) | 61.7 |
|  | 86 | 17,544 | 1.36  (0.29, 2.48) | -0.14  (-1.21, 0.98) | 1.35  (1.34, 1.36) | 28.0 |
| 20% | 6 | 6,938 | 1.18  (-0.76, 3.15) | -0.32  (-2.26, 1.65) | 1.19  (1.17, 1.21) | 77.5 |
|  | 86 | 17,544 | 1.20  (0.28, 2.16) | -0.30  (-1.22, 0.66) | 1.20  (1.19, 1.21) | 33.2 |

*Table legend:* This table shows the observed ARR (i.e., observed treatment effect) and bias (difference between observed treatment effect and true treatment effect). The true treatment effect is fixed at an ARR of 1.5% in all simulations. Patients in each group have an equal probability of being misclassified, with proportions set at the values specified in the left-most column (i.e., blinded trial simulation).

*Abbreviations:* ARR, absolute risk reduction; MCSE, Monte Carlo standard error; WLST, withdrawal of life-sustaining treatment

**eTable5: Bias from WLST when treatment leads to an ARR of 6% in poor outcomes**

| **Misclassified fraction** | **Control group poor outcome probability (%)** | **Sample size (n)** | **Observed ARR after WLST, (95% CI)** | **Bias, (95% CI)** | **MCSE** | **% trials with reversal of statistical interpretation** |
| --- | --- | --- | --- | --- | --- | --- |
|  |  |  |  |  |  |  |
| 2% | 37 | 1,954 | 5.89  (1.73, 10.08) | -0.11  (-4.27, 4.08) | 5.9  (5.85, 5.94) | 24.2 |
|  | 50 | 2,170 | 5.87  (1.71, 10.09) | -0.13  (-4.29, 4.09) | 5.87  (5.83, 5.91) | 22.4 |
|  | 63 | 2,090 | 5.87  (1.58, 10.03) | -0.13  (-4.42, 4.03) | 5.92  (5.88, 5.96) | 18.8 |
|  | 86 | 1,228 | 5.87  (1.73, 10.05) | -0.13  (-4.27, 4.05) | 5.88  (5.84, 5.92) | 19.2 |
| 5% | 37 | 1,954 | 5.71  (1.34, 9.99) | -0.29  (-4.66, 3.99) | 5.69  (5.65, 5.73) | 27.3 |
|  | 50 | 2,170 | 5.74  (1.51, 9.84) | -0.26  (-4.49, 3.84) | 5.71  (5.66, 5.75) | 24.1 |
|  | 63 | 2,090 | 5.72  (1.60, 9.83) | -0.28  (-4.40, 3.83) | 5.71  (5.67, 5.75) | 21.1 |
|  | 86 | 1,228 | 5.73  (1.65, 9.76) | -0.27  (-4.35, 3.76) | 5.73  (5.68, 5.77) | 20.2 |
| 10% | 37 | 1,954 | 5.37  (0.95, 9.71) | -0.63  (-5.05, 3.71) | 5.36  (5.32, 5.40) | 32.7 |
|  | 50 | 2,170 | 5.41  (1.27, 9.56) | -0.59  (-4.73, 3.56) | 5.41  (5.37, 5.45) | 28.7 |
|  | 63 | 2,090 | 5.38  (1.27, 9.52) | -0.62  (-4.73, 3.52) | 5.39  (5.35, 5.44) | 24.5 |
|  | 86 | 1,228 | 5.40  (1.32, 9.28) | -0.60  (-4.70, 3.28) | 5.42  (5.38, 5.46) | 23.6 |
| 20% | 37 | 1,954 | 4.81  (0.33, 9.24) | -1.19  (-5.67, 3.24) | 4.85  (4.81, 4.90) | 43.0 |
|  | 50 | 2,170 | 4.77  (0.67, 8.88) | -1.23  (-5.33, 2.88) | 4.80  (4.76, 4.85) | 37.5 |
|  | 63 | 2,090 | 4.86  (0.86, 8.85) | -1.14  (-5.14, 2.85) | 4.82  (4.78, 4.86) | 32.8 |
|  | 86 | 1,228 | 4.79  (0.98, 8.60) | -1.21  (-5.02, 2.60) | 4.78  (4.74, 4.81) | 28.9 |

*Table legend:* This table shows the observed ARR (i.e., observed treatment effect) and bias (difference between observed treatment effect and true treatment effect). The true treatment effect is fixed at an ARR of 6% in all simulations. Patients in each group have an equal probability of being misclassified, with proportions set at the values specified in the left-most column (i.e., blinded trial simulation).

*Abbreviations:* ARR, absolute risk reduction; MCSE, Monte Carlo standard error; WLST, withdrawal of life-sustaining treatment

**eTable6: Bias from WLST when treatment leads to an ARR of 10% in poor outcomes**

| **Misclassified fraction** | **Control group poor outcome probability (%)** | **Sample size (n)** | **Observed ARR after WLST, (95% CI)** | **Bias, (95% CI)** | **MCSE** | **% trials with reversal of statistical interpretation** |
| --- | --- | --- | --- | --- | --- | --- |
|  |  |  |  |  |  |  |
| 2% | 37 | 680 | 9.80  (2.79, 16.86) | -0.20  (-7.21, 6.86) | 9.76  (9.69, 9.83) | 19.3 |
|  | 50 | 774 | 9.80  (3.01, 16.69) | -0.20  (-6.99, 6.69) | 9.8  (9.73, 9.87) | 19.9 |
|  | 63 | 762 | 9.87  (2.86, 16.75) | -0.13  (-7.14, 6.75) | 9.87  (9.80, 9.94) | 20.7 |
|  | 86 | 480 | 9.85  (2.98, 16.73) | -0.15  (-7.02, 6.73) | 9.78  (9.71, 9.85) | 18.2 |
| 5% | 37 | 680 | 9.46  (2.34, 16.63) | -0.54  (-7.66, 6.63) | 9.55  (9.48, 9.62) | 25.8 |
|  | 50 | 774 | 9.46  (2.38, 16.27) | -0.54  (-7.62, 6.27) | 9.47  (9.40, 9.54) | 21.5 |
|  | 63 | 762 | 9.48  (2.54, 16.36) | -0.52  (-7.46, 6.36) | 9.45  (9.38, 9.52) | 23.5 |
|  | 86 | 480 | 9.46  (2.62, 16.32) | -0.54  (-7.38, 6.32) | 9.53  (9.46, 9.59) | 18.8 |
| 10% | 37 | 680 | 9.0  (1.76, 16.04) | -1.0  (-8.24, 6.04) | 9.04  (8.97, 9.11) | 32.4 |
|  | 50 | 774 | 8.97  (1.96, 15.99) | -1.03  (-8.04, 5.99) | 9.06  (8.99, 9.13) | 26.8 |
|  | 63 | 762 | 9.04  (2.15, 15.90) | -0.96  (-7.85, 5.90) | 9.02  (8.95, 9.09) | 27.1 |
|  | 86 | 480 | 8.93  (2.20, 15.55) | -1.07  (-7.80, 5.55) | 8.97  (8.91, 9.04) | 21.6 |
| 20% | 37 | 680 | 7.98  (0.51, 15.43) | -2.02  (-9.49, 5.43) | 7.97  (7.89, 8.04) | 45.4 |
|  | 50 | 774 | 8.09  (1.10, 15.19) | -1.91  (-8.90, 5.19) | 8.0  (7.93, 8.07) | 37.1 |
|  | 63 | 762 | 7.98  (1.36, 14.69) | -2.02  (-8.65, 4.69) | 7.97  (7.90, 8.04) | 34.6 |
|  | 86 | 480 | 8.03  (1.54, 14.31) | -1.97  (-8.46, 4.31) | 8.0  (7.93, 8.06) | 26.9 |

*Table legend:* This table shows the observed ARR (i.e., observed treatment effect) and bias (difference between observed treatment effect and true treatment effect). The true treatment effect is fixed at an ARR of 10% in all simulations. Patients in each group have an equal probability of being misclassified, with proportions set at the values specified in the left-most column (i.e., blinded trial simulation).

*Abbreviations:* ARR, absolute risk reduction; MCSE, Monte Carlo standard error; WLST, withdrawal of life-sustaining treatment

**eTable7: Bias from WLST when treatment leads to an ARR of 14% in poor outcomes**

| **Misclassified fraction** | **Control group poor outcome probability (%)** | **Sample size (n)** | **Observed ARR after WLST, (95% CI)** | **Bias, (95% CI)** | **MCSE** | **% trials with reversal of statistical interpretation** |
| --- | --- | --- | --- | --- | --- | --- |
|  |  |  |  |  |  |  |
| 2% | 37 | 334 | 13.77  (4.03, 23.80) | -0.23  (-9.97, 9.80) | 13.68  (13.58, 13.78) | 21.0 |
|  | 50 | 390 | 13.83  (3.90, 23.56) | -0.17  (-10.10, 9.56) | 13.72  (13.62, 13.81) | 23.3 |
|  | 63 | 392 | 13.21  (3.79, 23.14) | -0.79  (-10.21, 9.14) | 13.77  (13.67, 13.86) | 20.9 |
|  | 86 | 264 | 13.75  (4.55, 23.49) | -0.25  (-9.45, 9.49) | 13.81  (13.71, 13.90) | 19.7 |
| 5% | 37 | 334 | 13.08  (3.56, 22.08) | -0.92  (-10.44, 8.08) | 13.33  (13.23, 13.44) | 24.7 |
|  | 50 | 390 | 13.42  (3.50, 23.08) | -0.58  (-10.50, 9.08) | 13.37  (13.27, 13.46) | 26.7 |
|  | 63 | 392 | 13.54  (3.66, 23.36) | -0.46  (-10.34, 9.26) | 13.26  (13.16, 13.35) | 22.9 |
|  | 86 | 264 | 13.28  (3.53, 22.62) | -0.72  (-10.47, 8.62) | 13.25  (13.16, 13.35) | 21.4 |
| 10% | 37 | 334 | 12.54  (1.80, 22.85) | -1.46  (-12.20, 8.85) | 12.59  (12.49, 12.69) | 31.6 |
|  | 50 | 390 | 12.63  (2.85, 22.58) | -1.37  (-11.15, 8.58) | 12.62  (12.52, 12.72) | 32.4 |
|  | 63 | 392 | 12.67  (2.66, 22.46) | -1.33  (-11.34, 8.46) | 12.60  (12.51, 12.70) | 27.3 |
|  | 86 | 264 | 12.73  (3.30, 22.66) | -1.27  (-10.70, 8.66) | 12.52  (12.43, 12.61) | 25.4 |
| 20% | 37 | 334 | 11.1  (0.60, 21.63) | -2.9  (-13.40, 7.63) | 11.21  (11.10, 11.32) | 46.2 |
|  | 50 | 390 | 11.18  (1.61, 20.84) | -2.82  (-12.39, 6.84) | 11.30  (11.20, 11.40) | 41.7 |
|  | 63 | 392 | 11.30  (1.66, 20.92) | -2.70  (-12.34, 6.92) | 11.18  (11.08, 11.27) | 36.5 |
|  | 86 | 264 | 11.08  (2.33, 19.74) | -2.92  (-11.67, 5.74) | 11.27  (11.18, 11.36) | 30.4 |

*Table legend:* This table shows the observed ARR (i.e., observed treatment effect) and bias (difference between observed treatment effect and true treatment effect). The true treatment effect is fixed at an ARR of 14% in all simulations. Patients in each group have an equal probability of being misclassified, with proportions set at the values specified in the left-most column (i.e., blinded trial simulation).

*Abbreviations:* ARR, absolute risk reduction; MCSE, Monte Carlo standard error; WLST, withdrawal of life-sustaining treatment

**eTable8: Bias from WLST when treatment leads to an ARR of 17% in poor outcomes**

| **Misclassified fraction** | **Poor outcome probability in control group (%)** | **Sample size of the simulated study (n)** | **Observed ARR after WLST, (95% CI)** | **Bias, (95% CI)** | **MCSE** | **% trials with reversal of statistical interpretation** |
| --- | --- | --- | --- | --- | --- | --- |
|  |  |  |  |  |  |  |
| 2% | 37 | 218 | 16.57  (4.59, 29.41) | -0.43  (-12.41, 12.41) | 16.59  (16.47, 16.71) | 23.7 |
|  | 50 | 262 | 16.61  (4.92, 28.33) | -0.39  (-12.08, 11.33) | 16.74  (16.62, 16.86) | 23.6 |
|  | 63 | 268 | 16.79  (4.73, 28.15) | -0.21  (-12.27, 11.15) | 16.68  (16.56, 16.79) | 22.0 |
|  | 86 | 188 | 16.80  (5.16, 28.67) | -0.20  (-11.84, 11.67) | 16.63  (16.51, 16.75) | 20.2 |
| 5% | 37 | 218 | 15.91  (4.26, 27.83) | -1.09  (-12.74, 10.83) | 16.12  (16.00, 16.25) | 29.2 |
|  | 50 | 262 | 16.14  (4.80, 26.88) | -0.86  (-12.20, 9.88) | 16.05  (15.94, 16.17) | 24.5 |
|  | 63 | 268 | 16.23  (3.74, 27.67) | -0.77  (-13.26, 10.67) | 16.15  (16.03, 16.26) | 24.5 |
|  | 86 | 188 | 16.08  (4.58, 27.66) | -0.92  (-12.42, 10.66) | 16.09  (15.97, 16.20) | 22.1 |
| 10% | 37 | 218 | 15.24  (2.54, 27.81) | -1.76  (-14.46, 10.81) | 15.31  (15.18, 15.44) | 36.9 |
|  | 50 | 262 | 15.28  (3.43, 27.11) | -1.72  (-13.57, 10.11) | 15.28  (15.16, 15.40) | 31.4 |
|  | 63 | 268 | 15.47  (2.98, 26.82) | -1.53  (-14.02, 9.82) | 15.31  (15.20, 15.43) | 29.2 |
|  | 86 | 188 | 15.24  (3.89, 26.56) | -1.76  (-13.11, 9.56) | 15.31  (15.20, 15.43) | 25.8 |
| 20% | 37 | 218 | 13.69  (0.49, 26.54) | -3.31  (-16.51, 9.54) | 13.52  (13.39, 13.65) | 49.2 |
|  | 50 | 262 | 13.64  (1.46, 25.41) | -3.36  (-15.54, 8.41) | 13.52  (13.40, 13.64) | 40.9 |
|  | 63 | 268 | 13.77  (1.41, 25.02) | -3.23  (-15.59, 8.02) | 13.56  (13.45, 13.68) | 37.5 |
|  | 86 | 188 | 13.61  (2.95, 24.46) | -3.39  (-14.05, 7.46) | 13.63  (13.52, 13.74) | 32.4 |

*Table legend:* This table shows the observed ARR (i.e., observed treatment effect) and bias (difference between observed treatment effect and true treatment effect). The true treatment effect is fixed at an ARR of 17% in all simulations. Patients in each group have an equal probability of being misclassified, with proportions set at the values specified in the left-most column (i.e., blinded trial simulation).

*Abbreviations:* ARR, absolute risk reduction; MCSE, Monte Carlo standard error; WLST, withdrawal of life-sustaining treatment

**eTable9: Bias from WLST when treatment leads to an absolute risk increase by 5% in poor outcomes**

| **Misclassified fraction** | **Poor outcome probability in control group (%)** | **Sample size of the simulated study (n)** | **Observed ARI after WLST, (95% CI)** | **Bias, (95% CI)** | **MCSE** | **% trials with reversal of statistical interpretation** |
| --- | --- | --- | --- | --- | --- | --- |
|  |  |  |  |  |  |  |
| 2% | 37 | 1,426 | 4.87  (0.33, 10.00) | -0.13  (-5.33, 5.00) | -4.91  (-4.96, -4.86) | 53.7 |
|  | 50 | 1,592 | 4.89  (0.06, 9.84) | -0.11  (-4.94, 4.84) | -4.87  (-4.92, -4.83) | 48.3 |
|  | 63 | 1,542 | 4.92  (0.10, 9.62) | -0.08  (-4.90, 4.62) | -4.88  (-4.93, -4.84) | 47.7 |
|  | 86 | 922 | 4.89  (0.77, 8.97) | -0.11  (-4.23, 3.97) | -4.90  (-4.94, -4.86) | 32.4 |
| 5% | 37 | 1,426 | 4.75  (-0.26, 9.93) | 0.25  (-5.26, 4.93) | -4.73  (-4.78, -4.68) | 56.0 |
|  | 50 | 1,592 | 4.79  (-0.25, 9.68) | 0.21  (-4.68, 5.25) | -4.76  (-4.81, -4.72) | 50.3 |
|  | 63 | 1,542 | 4.80  (0.08, 9.50) | -0.20  (-4.92, 4.50) | -4.75  (-4.80, -4.71) | 49.2 |
|  | 86 | 922 | 4.75  (0.80, 8.71) | -0.25  (-4.20, 3.71) | -4.75  (-4.79, -4.71) | 34.7 |
| 10% | 37 | 1,426 | 4.53  (-0.62, 9.64) | -0.47  (-5.62, 4.64) | -4.50  (-4.55, -4.45) | 59.5 |
|  | 50 | 1,592 | 4.47  (-0.37, 9.31) | -0.53  (-5.37, 4.31) | -4.49  (-4.53, -4.44) | 54.8 |
|  | 63 | 1,542 | 4.50  (-0.02, 9.02) | -0.50  (-5.02, 4.02) | -4.48  (-4.52, -4.43) | 53.1 |
|  | 86 | 922 | 4.52  (0.57, 8.42) | -0.48  (-4.43, 3.42) | -4.49  (-4.53, -4.45) | 36.8 |
| 20% | 37 | 1,426 | 3.98  (-1.23, 9.26) | -1.02  (-6.23, 4.26) | -4.05  (-4.10, -4.0) | 67.0 |
|  | 50 | 1,592 | 3.99  (-0.79, 8.76) | -1.01  (-5.79, 3.76) | -4.0  (-4.05, -3.95) | 63.6 |
|  | 63 | 1,542 | 4.01  (-0.49, 8.82) | -0.99  (-5.49, 3.42) | -4.01  (-4.05, -3.96) | 59.3 |
|  | 86 | 922 | 3.98  (0.25, 7.61) | -1.02  (-4.75, 2.61) | -3.99  (-4.03, -3.95) | 41.3 |

*Table legend:* This table shows the observed ARI (i.e., observed treatment effect) and bias (difference between observed treatment effect and true treatment effect). The true treatment effect is fixed at an ARI of 5% in all simulations. Patients in each group have an equal probability of being misclassified, with proportions set at the values specified in the left-most column (i.e., blinded trial simulation).

*Abbreviations:* ARI, absolute risk increase; MCSE, Monte Carlo standard error; WLST, withdrawal of life-sustaining treatment

**eTable10: Bias from WLST when the ARR is 1.5% and misclassified fractions are imbalanced**

| **Misclassified fraction in treatment group (%)** | **Misclassified fraction in control group (%)** | **Control group poor outcome probability (%)** | **Sample size, (n)** | **Observed ARR after WLST,**  **(95% CI)** | **Bias,**  **(95% CI)** | **MCSE** | **% trials with reversal of statistical interpretation** |
| --- | --- | --- | --- | --- | --- | --- | --- |
|  |  |  |  |  |  |  |  |
| 1.6 | 2 | 6 | 6,938 | 1.84  (0.69, 3.07) | 0.34  (-0.81, 1.57) | 1.85  (1.84, 1.86) | 13.8 |
| 1.6 | 2 | 86 | 17,544 | 1.54  (0.54, 2.65) | 0.04  (-0.96, 1.15) | 1.54  (1.53, 1.55) | 19.8 |
| 4 | 5 | 6 | 6,938 | 2.41  (1.03, 3.82) | 0.91  (-0.47, 2.32) | 2.38  (2.37, 2.39) | 6.5 |
| 4 | 5 | 86 | 17,544 | 1.58  (0.51, 2.63) | 0.08  (-0.99, 1.13) | 1.58  (1.57, 1.59) | 15.2 |
| 8 | 10 | 86 | 17,544 | 1.67  (0.66, 2.64) | 0.17  (-0.84, 1.14) | 1.66  (1.65, 1.67) | 11.3 |
| 16 | 20 | 86 | 17,544 | 1.82  (0.85, 2.79) | 0.32  (-0.65, 1.29) | 1.82  (1.81, 1.83) | 4.0 |

*Table legend:* This table shows the observed ARR (i.e., observed treatment effect) and bias (difference between observed treatment effect and true treatment effect). The true treatment effect is fixed at an ARR of 1.5% in all simulations. Patients in the treated group have a 20% lower probability of being misclassified, reflecting potentially more cautious approaches to WLST due to belief in treatment efficacy (i.e., unblinded trial simulation). Misclassified fractions are set at the values specified in the two left most-columns.

*Abbreviations:* ARR, absolute risk reduction; MCSE, Monte Carlo standard error; WLST, withdrawal of life-sustaining treatment

**eTable11: Bias from WLST when the ARR is 6% and misclassified fractions are imbalanced**

| **Misclassified fraction in treatment group (%)** | **Misclassified fraction in control group (%)** | **Control group poor outcome probability (%)** | **Sample size, (n)** | **Observed ARR after WLST,**  **(95% CI)** | **Bias,**  **(95% CI)** | **MCSE** | **% trials with reversal of statistical interpretation** |
| --- | --- | --- | --- | --- | --- | --- | --- |
|  |  |  |  |  |  |  |  |
| 1.6 | 2 | 37 | 1,954 | 6.13  (1.82, 10.31) | 0.13  (-4.18, 4.31) | 6.17  (6.12, 6.21) | 20.8 |
| 1.6 | 2 | 50 | 2,170 | 6.12  (1.93, 10.28) | 0.12  (-4.07, 4.28) | 6.10  (6.06, 6.14) | 18.1 |
| 1.6 | 2 | 63 | 2,090 | 6.04  (1.82, 10.21) | 0.04  (-4.18, 4.21) | 6.05  (6.01, 6.09) | 16.3 |
| 1.6 | 2 | 86 | 1,228 | 5.94  (1.79, 10.11) | -0.06  (-4.21, 4.11) | 5.96  (5.92, 6.00) | 18.1 |
| 4 | 5 | 37 | 1,954 | 6.38  (2.11, 10.61) | 0.38  (-3.89, 4.61) | 6.41  (6.36, 6.45) | 18.0 |
| 4 | 5 | 50 | 2,170 | 6.25  (2.03, 10.43) | 0.25  (-3.97, 4.43) | 6.26  (6.22, 6.30) | 16.3 |
| 4 | 5 | 63 | 2,090 | 6.13  (1.94, 10.33) | 0.13  (-4.06, 4.33) | 6.13  (6.09, 6.17) | 14.7 |
| 4 | 5 | 86 | 1,228 | 5.90  (1.78, 10.08) | -0.10  (-4.22, 4.08) | 5.93  (5.89, 5.97) | 17.7 |
| 8 | 10 | 37 | 1,954 | 6.8  (2.35, 11.08) | 0.80  (-3.65, 5.08) | 6.76  (6.71, 6.80) | 13.9 |
| 8 | 10 | 50 | 2,170 | 6.53  (2.37, 10.75) | 0.53  (-3.63, 4.75) | 6.53  (6.49, 6.56) | 12.5 |
| 8 | 10 | 63 | 2,090 | 6.28  (2.16, 10.45) | 0.28  (-3.84, 4.45) | 6.27  (6.23, 6.31) | 14.1 |
| 8 | 10 | 86 | 1,228 | 5.82  (1.83, 9.84) | -0.18  (-4.17, 3.84) | 5.80  (5.76, 5.84) | 18.5 |
| 16 | 20 | 50 | 2,170 | 7.0  (2.89, 11.11) | 1.0  (-3.11, 5.11) | 6.99  (6.95, 7.03) | 8.1 |
| 16 | 20 | 63 | 2,090 | 6.5  (2.49, 10.49) | 0.50  (-3.51, 4.49) | 6.50  (6.46, 6.54) | 10.7 |
| 16 | 20 | 86 | 1,228 | 5.6  (1.80, 9.45) | -0.40  (-4.20, 3.45) | 5.60  (5.56, 5.64) | 16.4 |

*Table legend:* This table shows the observed ARR (i.e., observed treatment effect) and bias (difference between observed treatment effect and true treatment effect). The true treatment effect is fixed at an ARR of 6.0% in all simulations. Patients in the treated group have a 20% lower probability of being misclassified, reflecting potentially more cautious approaches to WLST due to belief in treatment efficacy (i.e., unblinded trial simulation). Misclassified fractions are set at the values specified in the two left most-columns.

*Abbreviations:* ARR, absolute risk reduction; MCSE, Monte Carlo standard error; WLST, withdrawal of life-sustaining treatment

**eTable12: Bias from WLST when the ARR is 10% and misclassified fractions are imbalanced**

| **Misclassified fraction in treatment group (%)** | **Misclassified fraction in control group (%)** | **Control group poor outcome probability (%)** | **Sample size, (n)** | **Observed ARR after WLST,**  **(95% CI)** | **Bias,**  **(95% CI)** | **MCSE** | **% trials with reversal of statistical interpretation** |
| --- | --- | --- | --- | --- | --- | --- | --- |
|  |  |  |  |  |  |  |  |
| 1.6 | 2 | 37 | 680 | 10.06  (2.74, 17.14) | 0.06  (-7.26, 7.14) | 10.12  (10.05, 10.19) | 18.7 |
| 1.6 | 2 | 50 | 774 | 10.01  (2.94, 17.06) | 0.01  (-7.06, 7.06) | 10.08  (10.01, 10.15) | 17.2 |
| 1.6 | 2 | 63 | 762 | 9.95  (3.08, 16.76) | -0.05  (-6.92, 6.76) | 9.95  (9.91, 9.99) | 20.4 |
| 1.6 | 2 | 86 | 480 | 9.91  (3.04, 16.67) | -0.09  (-6.96, 6.67) | 9.87  (9.80, 9.94) | 14.9 |
| 4 | 5 | 37 | 680 | 10.17  (3.03, 17.11) | 0.17  (-6.97, 7.11) | 10.2  (10.13, 10.27) | 18.9 |
| 4 | 5 | 50 | 774 | 10.05  (3.06, 16.98) | 0.05  (-6.94, 6.98) | 10.11  (10.04, 10.17) | 16.4 |
| 4 | 5 | 63 | 762 | 10.02  (2.95, 17.04) | 0.02  (-7.05, 7.04) | 9.95  (9.88, 10.02) | 20.1 |
| 4 | 5 | 86 | 480 | 9.79  (2.99, 16.59) | -0.21  (-7.01, 6.59) | 9.71  (9.65, 9.78) | 16.4 |
| 8 | 10 | 37 | 680 | 10.46  (3.23, 17.58) | 0.46  (-6.77, 7.58) | 10.38  (10.31, 10.46) | 18.5 |
| 8 | 10 | 50 | 774 | 10.13  (3.04, 17.09) | 0.13  (-6.96, 7.09) | 10.24  (10.17, 10.31) | 14.9 |
| 8 | 10 | 63 | 762 | 9.97  (3.09, 16.70) | -0.03  (-6.91, 6.70) | 9.94  (9.88, 10.01) | 19.6 |
| 8 | 10 | 86 | 480 | 9.51  (2.79, 16.35) | -0.49  (-7.21, 6.35) | 9.50  (9.43, 9.57) | 17.6 |
| 16 | 20 | 50 | 774 | 10.4  (3.44, 17.47) | 0.4  (-6.56, 7.47) | 10.4  (10.33, 10.47) | 16.0 |
| 16 | 20 | 63 | 762 | 9.90  (3.15, 16.67) | -0.10  (-6.85, 6.67) | 9.81  (9.74, 9.88) | 16.4 |
| 16 | 20 | 86 | 480 | 8.97  (2.49, 15.34) | -1.03  (-7.51, 5.34) | 8.96  (8.90, 9.03) | 16.8 |

*Table legend:* This table shows the observed ARR (i.e., observed treatment effect) and bias (difference between observed treatment effect and true treatment effect). The true treatment effect is fixed at an ARR of 10.0% in all simulations. Patients in the treated group have a 20% lower probability of being misclassified, reflecting potentially more cautious approaches to WLST due to belief in treatment efficacy (i.e., unblinded trial simulation). Misclassified fractions are set at the values specified in the two left most-columns.

*Abbreviations:* ARR, absolute risk reduction; MCSE, Monte Carlo standard error; WLST, withdrawal of life-sustaining treatment

**eTable13: Bias from WLST when the ARR is 14% and misclassified fractions are imbalanced**

*Table legend:* This table shows the observed ARR (i.e., observed treatment effect) and bias (difference between observed treatment effect and true treatment effect). The true treatment effect is fixed at an ARR of 14.0% in all simulations. Patients in the treated group have a 20% lower probability of being misclassified, reflecting potentially more cautious approaches to WLST due to belief in treatment efficacy (i.e., unblinded trial simulation). Misclassified fractions are set at the values specified in the two left most-columns.

*Abbreviations:* ARR, absolute risk reduction; MCSE, Monte Carlo standard error; WLST, withdrawal of life-sustaining treatment

| **Misclassified fraction in treatment group (%)** | **Misclassified fraction in control group (%)** | **Control group poor outcome probability (%)** | **Sample size, (n)** | **Observed ARR after WLST,**  **(95% CI)** | **Bias,**  **(95% CI)** | **MCSE** | **% trials with reversal of statistical interpretation** |
| --- | --- | --- | --- | --- | --- | --- | --- |
|  |  |  |  |  |  |  |  |
| 1.6 | 2 | 37 | 334 | 14.01  (4.25, 23.87) | 0.01  (-9.75, 9.87) | 14.06  (13.96, 14.16) | 18.0 |
| 1.6 | 2 | 50 | 390 | 13.93  (4.15, 23.52) | -0.07  (-9.85, 9.52) | 13.94  (13.84, 14.04) | 22.0 |
| 1.6 | 2 | 63 | 392 | 13.91  (4.34, 23.47) | -0.09  (-9.66, 9.47) | 13.95  (13.85, 14.05) | 20.3 |
| 1.6 | 2 | 86 | 264 | 13.90  (4.23, 23.47) | -0.10  (-9.77, 9.47) | 13.87  (13.77, 13.97) | 20.5 |
| 4 | 5 | 37 | 334 | 14.11  (3.84, 24.10) | 0.11  (-10.16, 10.10) | 14.06  (13.96, 14.16) | 21.1 |
| 4 | 5 | 50 | 390 | 13.96  (4.17, 23.95) | -0.04  (-9.83, 9.95) | 13.89  (13.80, 13.99) | 22.9 |
| 4 | 5 | 63 | 392 | 13.74  (4.15, 23.34) | -0.26  (-9.85, 9.34) | 13.83  (13.73, 13.93) | 20.5 |
| 4 | 5 | 86 | 264 | 13.56  (4.09, 23.16) | -0.44  (-9.91, 9.16) | 13.55  (13.46, 13.65) | 20.9 |
| 8 | 10 | 37 | 334 | 14.18  (3.92, 24.31) | 0.18  (-10.08, 10.31) | 14.03  (13.93, 14.13) | 21.6 |
| 8 | 10 | 50 | 390 | 13.84  (3.67, 23.42) | -0.16  (-10.33, 9.42) | 13.79  (13.69, 13.88) | 22.9 |
| 8 | 10 | 63 | 392 | 13.63  (3.95, 23.12) | -0.37  (-10.05, 9.12) | 13.63  (13.53, 13.73) | 20.9 |
| 8 | 10 | 86 | 264 | 13.11  (3.77, 22.26) | -0.89  (-10.23, 8.26) | 13.15  (13.06, 13.24) | 21.2 |
| 16 | 20 | 50 | 390 | 13.74  (3.96, 23.52) | -0.26  (-10.04, 9.52) | 13.75  (13.65, 13.85) | 23.6 |
| 16 | 20 | 63 | 392 | 13.23  (3.62, 22.80) | -0.77  (-10.38, 8.80) | 13.23  (13.13, 13.32) | 20.8 |
| 16 | 20 | 86 | 264 | 12.41  (3.36, 21.28) | -1.59  (-10.64, 7.28) | 12.37  (12.28, 12.46) | 23.2 |

**eTable14: Bias from WLST when the ARR is 17% and misclassified fractions are imbalanced**

| **Misclassified fraction in treatment group (%)** | **Misclassified fraction in control group (%)** | **Control group poor outcome probability (%)** | **Sample size, (n)** | **Observed ARR after WLST,**  **(95% CI)** | **Bias,**  **(95% CI)** | **MCSE** | **% trials with reversal of statistical interpretation** |
| --- | --- | --- | --- | --- | --- | --- | --- |
|  |  |  |  |  |  |  |  |
| 1.6 | 2 | 37 | 218 | 16.98  (4.78, 28.97) | -0.02  (-12.22, 11.97) | 16.83  (16.71, 16.95) | 22.9 |
| 1.6 | 2 | 50 | 262 | 16.94  (5.19, 28.50) | -0.06  (-11.81, 11.50) | 16.90  (16.78, 17.02) | 21.2 |
| 1.6 | 2 | 63 | 268 | 16.82  (5.10, 28.54) | -0.18  (-11.90, 11.54) | 16.91  (16.80, 17.03) | 21.9 |
| 1.6 | 2 | 86 | 188 | 16.72  (4.92, 28.46) | -0.28  (-12.08, 11.46) | 16.73  (16.61, 16.85) | 19.5 |
| 4 | 5 | 37 | 218 | 17.04  (4.74, 28.93) | 0.04  (-12.26, 11.93) | 17.01  (16.89, 17.13) | 25.5 |
| 4 | 5 | 50 | 262 | 16.89  (4.90, 28.76) | -0.11  (-12.10, 11.76) | 16.85  (16.73, 16.97) | 22.1 |
| 4 | 5 | 63 | 268 | 16.81  (4.97, 28.70) | -0.19  (-12.03, 11.70) | 16.67  (16.56, 16.79) | 21.9 |
| 4 | 5 | 86 | 188 | 16.47  (4.62, 28.43) | -0.53  (-12.38, 11.43) | 16.49  (16.38, 16.61) | 19.1 |
| 8 | 10 | 37 | 218 | 16.93  (4.58, 29.51) | -0.07  (-12.42, 12.51) | 16.84  (16.71, 16.96) | 27.2 |
| 8 | 10 | 50 | 262 | 16.66  (4.58, 28.49) | -0.34  (-12.42, 11.49) | 16.66  (16.54, 16.78) | 22.5 |
| 8 | 10 | 63 | 268 | 16.31  (4.39, 28.14) | -0.69  (-12.61, 11.14) | 16.32  (16.21, 16.44) | 22.4 |
| 8 | 10 | 86 | 188 | 15.92  (4.73, 27.13) | -1.08  (-12.27, 10.13) | 15.80  (15.69, 15.91) | 21.5 |
| 16 | 20 | 50 | 262 | 16.33  (4.36, 28.57) | -0.67  (-12.64, 11.57) | 16.23  (16.11, 16.35) | 24.8 |
| 16 | 20 | 63 | 268 | 15.66  (4.13, 27.07) | -1.34  (-12.87, 10.07) | 15.80  (15.68, 15.91) | 24.4 |
| 16 | 20 | 86 | 188 | 14.83  (4.09, 25.99) | -2.17  (-12.91, 8.99) | 14.81  (14.70, 14.92) | 24.0 |

*Table legend:* This table shows the observed ARR (i.e., observed treatment effect) and bias (difference between observed treatment effect and true treatment effect). The true treatment effect is fixed at an ARR of 17.0% in all simulations. Patients in the treated group have a 20% lower probability of being misclassified, reflecting potentially more cautious approaches to WLST due to belief in treatment efficacy (i.e., unblinded trial simulation). Misclassified fractions are set at the values specified in the two left most-columns.

*Abbreviations:* ARR, absolute risk reduction; MCSE, Monte Carlo standard error; WLST, withdrawal of life-sustaining treatment

**eFigure1: Bias from WLST when treatment has no effect (i.e., ARR 0%) and misclassified fractions are imbalanced**


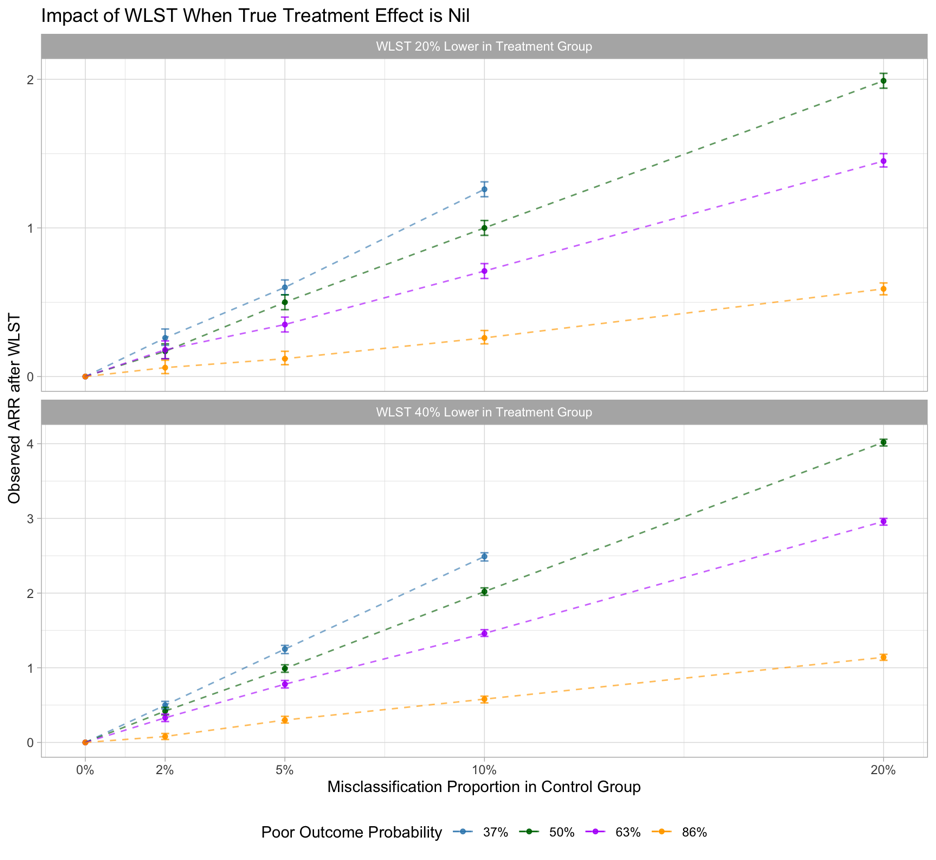


*Figure Legend:* This figure shows the impact of WLST misclassification on the observed treatment effect for a treatment where the true effect is nil (i.e., ARR=0%). Two different misclassified fractions are shown (20% and 40%), reflecting different levels of clinicians’ belief in the treatment. In all simulations, WLST misclassification leads to the perception of treatment benefit based on the average observed treatment effect (individual dots), with greater benefit for lower levels of illness severity and higher levels of misclassification. The error bars around each dot represent Monte Carlo simulation-related variability.

*Abbreviations:* ARR, absolute risk reduction; WLST, withdrawal of life-sustaining treatment

**eTable15: Bias from WLST when treatment has no effect (i.e., ARR 0%) and misclassified fractions are imbalanced**

| **Misclassified fraction in treatment group (%)** | **Misclassified fraction in control group (%)** | **Control group poor outcome probability (%)** | **Sample size, (n)** | **Observed ARR after WLST,**  **(95% CI)** | **MCSE** | **% trials with reversal of statistical interpretation** | |
| --- | --- | --- | --- | --- | --- | --- | --- |
|  |  |  |  |  |  | **Appear effective** | **Appear harmful** |
|  |  |  |  |  |  |  |  |
| 1.6 | 2 | 37 | 1,426 | 0.25  (-4.75, 5.30) | 0.26  (0.21, 0.32) | 3.7 | 1.3 |
| 1.2 | 2 | 37 | 1,426 | 0.54  (-4.55, 5.59) | 0.50  (0.45, 0.55) | 4.1 | 0.9 |
| 1.6 | 2 | 50 | 1,592 | 0.14  (-4.88, 4.94) | 0.17  (0.12, 0.22) | 3.3 | 2.4 |
| 1.2 | 2 | 50 | 1,592 | 0.38  (-4.39, 5.34) | 0.42  (0.37, 0.47) | 4.8 | 1.6 |
| 1.6 | 2 | 63 | 1,542 | 0.18  (-4.62, 5.07) | 0.10  (0.06, 0.15) | 3.3 | 2.8 |
| 1.2 | 2 | 63 | 1,542 | 0.28  (-4.49, 5.15) | 0.33  (0.28, 0.38) | 3.9 | 2.5 |
| 1.6 | 2 | 86 | 922 | 0.06  (-4.45, 4.51) | 0.06  (0.02, 0.11) | 1.7 | 2.9 |
| 1.2 | 2 | 86 | 922 | 0.08  (-4.45, 4.57) | 0.08  (0.04, 0.12) | 1.7 | 2.9 |
| 4 | 5 | 37 | 1,426 | 0.65  (-4.47, 5.68) | 0.6  (0.55, 0.65) | 4.5 | 0.9 |
| 3 | 5 | 37 | 1,426 | 1.32  (-3.81, 6.41) | 1.25  (1.19, 1.30) | 7.5 | 0.8 |
| 4 | 5 | 50 | 1,592 | 0.49  (-4.50, 5.47) | 0.50  (0.45, 0.55) | 4.7 | 1.1 |
| 3 | 5 | 50 | 1,592 | 1.03  (-3.83, 5.91) | 0.99  (0.94, 1.04) | 6.4 | 0.7 |
| 4 | 5 | 63 | 1,542 | 0.35  (-4.49, 5.08) | 0.35  (0.30, 0.40) | 3.6 | 2.1 |
| 3 | 5 | 63 | 1,542 | 0.72  (-4.11, 5.46) | 0.78  (0.73, 0.83) | 4.7 | 1.3 |
| 4 | 5 | 86 | 922 | 0.14  (-4.29, 4.61) | 0.12  (0.08, 0.17) | 1.9 | 2.4 |
| 3 | 5 | 86 | 922 | 0.29  (-4.11, 4.75) | 0.30  (0.26, 0.35) | 2.3 | 2.4 |
| 8 | 10 | 37 | 1,426 | 1.23  (-4.03, 6.45) | 1.26  (1.21, 1.31) | 6.9 | 0.7 |
| 6 | 10 | 37 | 1,426 | 2.53  (-2.75, 7.66) | 2.49  (2.43, 2.54) | 15.9 | 0.1 |
| 8 | 10 | 50 | 1,592 | 0.98  (-3.88, 5.86) | 1.0  (0.95, 1.05) | 6.8 | 0.7 |
| 6 | 10 | 50 | 1,592 | 2.02  (-2.89, 6.93) | 2.02  (1.97, 2.07) | 13.2 | 0.3 |
| 8 | 10 | 63 | 1,542 | 0.75  (-3.95, 5.48) | 0.71  (0.66, 0.76) | 5.3 | 1.2 |
| 6 | 10 | 63 | 1,542 | 1.45  (-3.29, 6.11) | 1.46  (1.42, 1.51) | 9.3 | 0.4 |
| 8 | 10 | 86 | 922 | 0.28  (-4.12, 4.56) | 0.26  (0.22, 0.31) | 2.1 | 2.5 |
| 6 | 10 | 86 | 922 | 0.55  (-3.70, 4.94) | 0.58  (0.53, 0.62) | 3.2 | 2.0 |
| 16 | 20 | 50 | 1,592 | 1.99  (-2.82, 6.82) | 1.99  (1.94, 2.04) | 14.9 | 0.1 |
| 12 | 20 | 50 | 1,592 | 4.01  (-0.82, 8.89) | 4.02  (3.97, 4.06) | 37.5 | 0.0 |
| 16 | 20 | 63 | 1,542 | 1.49  (-3.12, 6.07) | 1.45  (1.41, 1.50) | 9.7 | 0.7 |
| 12 | 20 | 63 | 1,542 | 2.97  (-1.56, 7.52) | 2.96  (2.91, 3.00) | 24.4 | 0.0 |
| 16 | 20 | 86 | 922 | 0.56  (-3.57, 4.68) | 0.59  (0.55, 0.63) | 3.9 | 1.5 |
| 12 | 20 | 86 | 922 | 1.18  (-2.92, 5.32) | 1.14  (1.10, 1.18) | 7.2 | 1.1 |

*Table legend:* This table shows the observed ARR (i.e., observed treatment effect) and bias (difference between observed treatment effect and true treatment effect). The true treatment has no effect and therefore an ARR of 0% is used in all simulations. For this particular analysis, patients in the treated group have either a 20% or 40% lower probability of being misclassified, reflecting different approaches to WLST due to varying strengths of belief in treatment efficacy (i.e., unblinded trial simulation). Misclassified fractions are set at the values specified in the two left most-columns.

*Abbreviations:* ARR, absolute risk increase; MCSE, Monte Carlo standard error; WLST, withdrawal of life-sustaining treatment

**eTable16: Bias from WLST when absolute risk increase in poor outcomes is 5% and misclassified fractions are imbalanced**

| **Misclassified fraction in treatment group (%)** | **Misclassified fraction in control group (%)** | **Control group poor outcome probability (%)** | **Sample size, (n)** | **Observed ARR after WLST,**  **(95% CI)** | **Bias,**  **(95% CI)** | **MCSE** | **% trials with reversal of statistical interpretation** |
| --- | --- | --- | --- | --- | --- | --- | --- |
|  |  |  |  |  |  |  |  |
| 1.6 | 2 | 37 | 1,426 | 4.67  (-0.33, 9.73) | -0.33  (-4.73, 5.33) | -4.67  (-4.72, -4.62) | 56.1 |
| 1.6 | 2 | 50 | 1,592 | 4.71  (-0.31, 9.68) | -0.29  (-4.68, 5.31) | -4.76  (-4.81, -4.71) | 49.6 |
| 1.6 | 2 | 63 | 1,542 | 4.76  (0.14, 947) | -0.24  (-4.47, 4.86) | -4.76  (-4.81, -4.72) | 49.7 |
| 1.6 | 2 | 86 | 922 | 4.90  (0.85, 8.96) | -0.10  (-4.15, 3.96) | -4.84  (-4.88, -4.80) | 32.3 |
| 4 | 5 | 37 | 1,426 | 4.19  (-9.35, 0.92) | 0.81  (-4.35, 5.92) | -4.20  (-4.25, -4.15 | 63.7 |
| 4 | 5 | 50 | 1,592 | 4.32  (-0.57, 9.30) | -0.68  (-5.57, 4.30) | -4.30  (-4.35, -4.25) | 56.5 |
| 4 | 5 | 63 | 1,542 | 4.42  (-0.34, 9.12) | -0.58  (-5.34, 4.12) | -4.43  (-4.47, -4.38) | 53.7 |
| 4 | 5 | 86 | 922 | 4.67  (0.71, 8.57) | -0.33  (-4.29, 3.57) | -4.64  (-4.68, -4.60) | 34.9 |
| 8 | 10 | 37 | 1,426 | 3.36  (-1.84, 8.51) | -1.64  (-6.84, 3.51) | -3.37  (-3.42, -3.31) | 75.2 |
| 8 | 10 | 50 | 1,592 | 3.57  (-1.24, 8.41) | -1.43  (-6.24, 3.41) | -3.62  (-3.67, -3.58) | 67.7 |
| 8 | 10 | 63 | 1,542 | 3.86  (-0.80, 8.45) | -1.14  (-5.80, 3.45) | -3.91  (-3.96, -3.86) | 62.7 |
| 8 | 10 | 86 | 922 | 4.36  (0.40, 8.39) | -0.64  (-4.60, 3.39) | -4.33  (-4.37, -4.29) | 38.1 |
| 16 | 20 | 50 | 1,592 | 2.2  (-2.57, 7.02) | -2.8  (-7.57, 2.02) | -2.21  (-2.26, -2.16) | 85.5 |
| 16 | 20 | 63 | 1,542 | 2.72  (-1.87, 7.22) | -2.28  (-6.87, 2.22) | -2.71  (-2.75, -2.66) | 77.3 |
| 16 | 20 | 86 | 922 | 3.61  (-0.10, 7.41) | -1.39  (-5.10, 2.41) | -3.64  (-3.68, -3.61) | 48.9 |

*Table legend:* This table shows the observed ARI (i.e., observed treatment effect) and bias (difference between observed treatment effect and true treatment effect). The true treatment effect is fixed at an ARI of 5.0% in all simulations. Patients in the treated group have a 20% lower probability of being misclassified, reflecting potentially more cautious approaches to WLST due to belief in treatment efficacy (i.e., unblinded trial simulation). Misclassified fractions are set at the values specified in the two left most-columns.

*Abbreviations:* ARI, absolute risk increase; MCSE, Monte Carlo standard error; WLST, withdrawal of life-sustaining treatment

**eTable17: Impact of WLST for effective treatments when the outcome is on an ordinal scale**

| ***Scenario 1: Simulating a transfusion trial*** | | | | ***Scenario 2: Simulating a thrombectomy trial*** | | | |
| --- | --- | --- | --- | --- | --- | --- | --- |
| **True pOR** | **WLST in treatment group (%)** | **WLST in control group (%)** | **Observed pOR**  **(95% CI)** | **True pOR** | **WLST in treatment group (%)** | **WLST in control group (%)** | **Observed pOR**  **(95% CI)** |
|  |  |  |  |  |  |  |  |
| Misclassified fraction balanced between groups | | | | | | | |
| 1.37  (1.08, 1.73) | 2 | 2 | 1.36  (1.09, 1.74) | 2.70  (1.68, 4.34) | 2 | 2 | 2.24  (1.39, 3.49) |
|  | 5 | 5 | 1.35  (1.04, 1.73) |  | 5 | 5 | 2.25  (1.42, 3.93) |
|  | 10 | 10 | 1.33  (1.03, 1.71) |  | 10 | 10 | 2.20  (1.39, 3.44) |
|  | 20 | 20 | 1.28  (0.99, 1.60) |  | 20 | 20 | 2.15  (1.38, 3.38) |
| Misclassified fraction lower in treatment group | | | | | | | |
| 1.37  (1.08, 1.73) | 1.6 | 2 | 1.37  (1.08, 1.73) | 2.70  (1.68, 4.34) | 1.6 | 2 | 2.46  (1.53, 3.89) |
|  | 4 | 5 | 1.38  (1.07, 1.74) |  | 4 | 5 | 2.39  (1.51, 3.65) |
|  | 8 | 10 | 1.38  (1.08, 1.78) |  | 8 | 10 | 2.34  (1.65, 3.24) |
|  | 16 | 20 | 1.39  (1.09, 1.79) |  | 16 | 20 | 2.29  (1.66, 3.10) |
|  |  |  |  |  |  |  |  |

*Table legend:* This table indicates the observed proportional odds ratios from two different trials after WLST misclassification. In scenario 1, we studied a blood transfusion trial in patients with acute brain injury that showed a modest benefit with transfusion (TRAIN trial). In scenario 2, we studied a thrombectomy trial in patients with basilar stroke that showed a significant benefit with thrombectomy (BAOCHE trial). The outcome in both trials is on an ordinal scale with eight and six levels, respectively. Control group probabilities within each ordinal grouping were taken from the respective published trials; sample sizes were also matched to their counterparts (n=850 and n=217, respectively). The outcome is expressed in terms of a “good neurologic outcome”. The true pOR was defined for each trial using the published trial results. Confidence intervals represent the uncertainty around the true pOR (these may be slightly different from the published trials due to simulation variability). Two different trial designs were studied. In the first, we kept the misclassified fraction the same between treatment and control groups (i.e., simulation of a blinded trial). In the second, the misclassified fraction was 20% lower in the treatment group compared to the control group (i.e., simulation of an unblinded trial). Misclassified fractions in each group are listed in table. For both trials, in the blinded scenario, the true treatment effect attenuated as the misclassified fraction increased (observed pORs became smaller than the true pOR), but the attenuation was modest in the transfusion trial. On the other hand, in the unblinded trial scenario, the observed treatment effect modestly increased as misclassified fraction increased in the transfusion trial scenario, but there was persistent attenuation in the case of the stroke trial, suggesting modulation of the observed effect by the strength of the true effect.

*Abbreviations:* pOR, proportional odds ratio; WLST, withdrawal of life-sustaining treatment

**eTable18: Impact of WLST for a neutral and harmful treatment when the outcome is on an ordinal scale**

| ***Scenario 3: Simulating a neutral treatment*** | | | | ***Scenario 4: Simulating a harmful treatment*** | | | |
| --- | --- | --- | --- | --- | --- | --- | --- |
| **True pOR** | **WLST in treatment group (%)** | **WLST in control group (%)** | **Observed pOR**  **(95% CI)** | **True pOR** | **WLST in treatment group (%)** | **WLST in control group (%)** | **Observed pOR**  **(95% CI)** |
|  |  |  |  |  |  |  |  |
| 1.00  (0.79, 1.27) | 1.6 | 2 | 1.01  (0.78, 1.27) |  | 1.6 | 2 | 0.82  (0.67, 1.01) |
|  | 4 | 5 | 1.02  (0.79, 1.29) | 0.80  (0.65, 0.96) | 4 | 5 | 0.83  (0.68, 1.02) |
|  | 8 | 10 | 1.04  (0.81, 1.29) |  | 8 | 10 | 0.85  (0.69, 1.04) |
|  | 16 | 20 | 1.35  (1.05, 1.71) |  | 16 | 20 | 0.89  (0.71, 1.09) |
|  |  |  |  |  |  |  |  |

*Table legend:* This table indicates the observed proportional odds ratios from two different hypothetical trials after WLST misclassification. The outcome in both trials is on an ordinal scale with eight categories. Control group probabilities within each ordinal grouping were taken from the TRAIN trial, which was a trial of blood transfusion in patients with acute brain injury. The sample size in the simulation was matched to the sample size of TRAIN (n=850). The rationale for using this trial is that it showed a small benefit with blood transfusion, and sample sizes for new interventions with unknown treatment effect sizes are usually powered with the presumption of benefit. The outcome is expressed in terms of a “good neurologic outcome”. Simulations mimicked an unblinded trial in which the WLST misclassified fraction was lower in the treatment group compared to the control group. Misclassified fractions in each group are listed in the table. For a neutral treatment (true pOR = 1), unblinding led to the perception of benefit based on the point estimate of the average observed treatment effect (pOR > 1), but confidence intervals were wide. For the harmful treatment (true pOR = 0.80), unblinding led to attenuation of harm (point estimates of the average observed treatment effect are closer to the null than the true treatment effect), but confidence intervals were wide.

*Abbreviations:* pOR, proportional odds ratio; WLST, withdrawal of life sustaining treatment

**eTable19: Bias correction using IPCW**

**
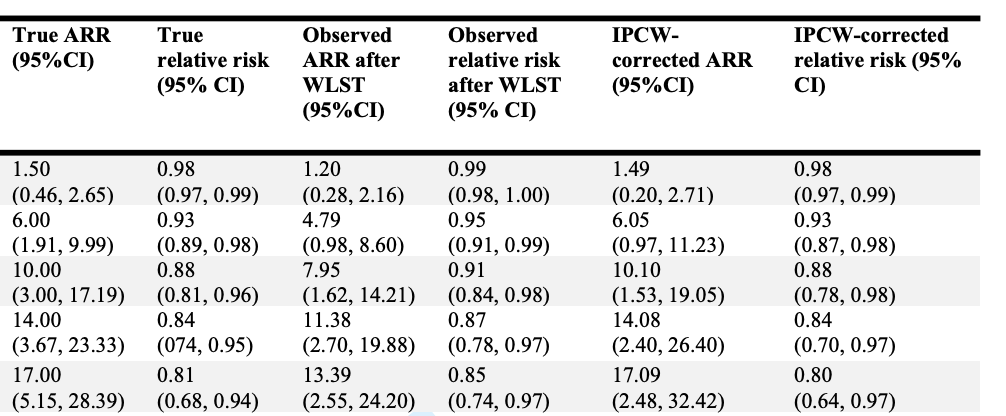
**

*Table Legend:* This table reports the use of IPCW to correct WLST-related bias. We repeated simulations from the main analysis in which the poor outcome probability was 86% and the WLST misclassification fraction was 20%. We chose these particular combinations because they led to the highest bias in the main analysis. Corresponding sample sizes were calculated using ARR values of 1.5%, 6%, 10%, 14%, and 17%. Confidence intervals for the true treatment effect were generated using bootstrap with 1,000 replicates. Note that some values of the observed ARR may be slightly different from the corresponding simulations presented earlier despite the same simulation inputs. This is because of sampling variability related to conducting the simulations more than once.

*Abbreviations:* ARR, absolute risk reduction; IPCW, inverse probability censoring weighting; WLST, withdrawal of life-sustaining treatment

**eTable20: Post-hoc analysis with 40% lower WLST misclassified fraction in the treated group (all simulations assume 50% baseline poor outcome probability)**

| **True treatment effect (ARR)** | **Misclassified fraction in treatment group (%)** | **Misclassified fraction in control group (%)** | **Sample size, (n)** | **Observed ARR after WLST,**  **(95% CI)** | **Bias,**  **(95% CI)** | **MCSE** | **% trials with reversal of statistical interpretation** |
| --- | --- | --- | --- | --- | --- | --- | --- |
|  |  |  |  |  |  |  |  |
| 6% | 1.2 | 2 | 2,170 | 6.33  (2.15, 10.40) | 0.33  (-3.85, 4.40) | 6.33  (6.29, 6.38) | 16.3 |
|  | 3 | 5 | 2,170 | 6.80  (2.57, 11.06) | 0.80  (-3.43, 5.06) | 6.80  (6.76, 6.84) | 11.9 |
|  | 6 | 10 | 2,170 | 7.66  (3.43, 11.82) | 1.66  (-2.57, 5.82) | 7.66  (7.62, 7.70) | 5.2 |
|  | 12 | 20 | 2,170 | 9.27  (5.06, 13.44) | 3.27  (-0.94, 7.44) | 9.27  (9.23, 9.31) | 0.70 |
| 10% | 1.2 | 2 | 774 | 10.28  (3.29, 17.16) | 0.28  (-6.71, 7.16) | 10.28  (10.21, 10.35) | 15.6 |
|  | 3 | 5 | 774 | 10.67  (3.69, 17.70) | 0.67  (-6.31, 7.70) | 10.67  (10.60, 10.74) | 13.8 |
|  | 6 | 10 | 774 | 11.42  (4.19, 18.37) | 1.42  (-5.81, 8.37) | 11.42  (11.34, 11.49) | 9.6 |
|  | 12 | 20 | 774 | 12.78  (5.71, 19.79) | 2.78  (-4.29, 9.79) | 12.78  (12.71, 12.85) | 3.6 |
| 14% | 1.2 | 2 | 390 | 14.32  (4.29, 23.89) | 0.32  (-9.71, 9.89) | 14.32  (14.22, 14.42) | 19.6 |
|  | 3 | 5 | 390 | 14.52  (4.82, 24.29) | 0.52  (-9.18, 10.29) | 14.52  (14.42, 14.62) | 18.2 |
|  | 6 | 10 | 390 | 15.16  (5.22, 25.04) | 1.16  (-8.78, 11.04) | 15.16  (15.07, 15.26) | 14.8 |
|  | 12 | 20 | 390 | 16.3  (6.51, 26.36) | 2.30  (-7.49, 12.36) | 16.30  (16.20, 16.40) | 11.0 |
| 17% | 1.2 | 2 | 262 | 17.19  (5.42, 28.74) | 0.19  (-11.58, 11.74) | 17.19  (17.07, 17.30) | 20.6 |
|  | 3 | 5 | 262 | 17.51  (5.79, 29.01) | 0.51  (-11.21, 12.01) | 17.51  (17.40, 17.63) | 19.4 |
|  | 6 | 10 | 262 | 17.85  (5.63, 29.71) | 0.85  (-11.37, 12.71) | 17.85  (17.73, 17.97) | 17.1 |
|  | 12 | 20 | 262 | 18.95  (18.83, 19.07) | 1.95  (-10.10, 13.79) | 18.95  (6.90, 30.79) | 12.6 |

*Table Legend:* This table shows the observed ARR (i.e., observed treatment effect) and bias (difference between observed treatment effect and true treatment effect). For simplicity, the poor outcome probability is fixed at 50% in all simulations. Patients in the treated group have a 40% lower probability of being misclassified, reflecting a significantly more cautious approaches to WLST due to strong belief in treatment efficacy (i.e., unblinded trial simulation). Misclassified fractions are set at the values specified in the two left most-columns.

*Abbreviations:* ARR, absolute risk reduction; MCSE, Monte Carlo standard error; WLST, withdrawal of life-sustaining treatment

**References**

1. Morris TP, White IR, Crowther MJ. Using simulation studies to evaluate statistical methods. *Stat Med.* 2019;38(11):2074-2102.

2. Nichol G, Leroux B, Wang H, et al. Trial of Continuous or Interrupted Chest Compressions during CPR. *New England Journal of Medicine.* 2015;373(23):2203-2214.

3. Liang F, Wu Y, Wang X, et al. General Anesthesia vs Conscious Sedation for Endovascular Treatment in Patients With Posterior Circulation Acute Ischemic Stroke: An Exploratory Randomized Clinical Trial. *JAMA Neurology.* 2023;80(1):64-72.

4. Søndergaard L, Kasner SE, Rhodes JF, et al. Patent Foramen Ovale Closure or Antiplatelet Therapy for Cryptogenic Stroke. *New England Journal of Medicine.* 2017;377(11):1033-1042.

5. Perkins GD, Ji C, Deakin CD, et al. A Randomized Trial of Epinephrine in Out-of-Hospital Cardiac Arrest. *New England Journal of Medicine.* 2018;379(8):711-721.

6. Nordberg P, Taccone FS, Truhlar A, et al. Effect of Trans-Nasal Evaporative Intra-arrest Cooling on Functional Neurologic Outcome in Out-of-Hospital Cardiac Arrest: The PRINCESS Randomized Clinical Trial. *JAMA.* 2019;321(17):1677-1685.

7. Liu X, Dai Q, Ye R, et al. Endovascular treatment versus standard medical treatment for vertebrobasilar artery occlusion (BEST): an open-label, randomised controlled trial. *The Lancet Neurology.* 2020;19(2):115-122.

8. Effects of tranexamic acid on death, disability, vascular occlusive events and other morbidities in patients with acute traumatic brain injury (CRASH-3): a randomised, placebo-controlled trial. *Lancet.* 2019;394(10210):1713-1723.

9. Elmer J, Torres C, Aufderheide TP, et al. Association of early withdrawal of life-sustaining therapy for perceived neurological prognosis with mortality after cardiac arrest. *Resuscitation.* 2016;102:127-135.

10. May TL, Ruthazer R, Riker RR, et al. Early withdrawal of life support after resuscitation from cardiac arrest is common and may result in additional deaths. *Resuscitation.* 2019;139:308-313.

11. Sanders WR, Barber JK, Temkin NR, et al. Recovery Potential in Patients Who Died After Withdrawal of Life-Sustaining Treatment: A TRACK-TBI Propensity Score Analysis. *Journal of Neurotrauma.* 2024.

12. Taccone FS, Rynkowski CB, Møller K, et al. Restrictive vs Liberal Transfusion Strategy in Patients With Acute Brain Injury: The TRAIN Randomized Clinical Trial. *Jama.* 2024;332(19):1623-1633.

13. Jovin TG, Li C, Wu L, et al. Trial of Thrombectomy 6 to 24 Hours after Stroke Due to Basilar-Artery Occlusion. *N Engl J Med.* 2022;387(15):1373-1384.
